# Serine synthesis pathway regulates cardiac differentiation from human pluripotent stem cells

**DOI:** 10.1016/j.isci.2025.112843

**Published:** 2025-06-07

**Authors:** Tomohiko C. Umei, Shugo Tohyama, Yuika Morita-Umei, Manami Katoh, Seitaro Nomura, Kotaro Haga, Takako Hishiki, Tomomi Matsuura, Hidenori Tani, Yusuke Soma, Otoya Sekine, Masatoshi Ohno, Masashi Nakamura, Taijun Moriwaki, Yoshikazu Kishino, Keiichi Fukuda, Masaki Ieda

**Affiliations:** 1 Department of Cardiology, Keio University School of Medicine, Tokyo, Japan; 2 Center for Preventive Medicine, Keio University School of Medicine, Tokyo, Japan; 3 Fujita Medical Innovation Center Tokyo, Fujita Health University, Tokyo, Japan; 4 Kanagawa Institute of Industrial Science and Technology (KISTEC), Kawasaki, Kanagawa, Japan; 5 Keio University Regenerative Medicine Research Center, Kanagawa, Japan; 6 Department of Cardiovascular Medicine, Graduate School of Medicine, University of Tokyo, Tokyo, Japan; 7 Department of Frontier Cardiovascular Science, Graduate School of Medicine, University of Tokyo, Tokyo, Japan; 8 Department of Biochemistry, Keio University School of Medicine, Tokyo, Japan; 9 Clinical and Translational Research Center, Keio University Hospital, Tokyo, Japan; 10 Department of Cardiovascular Surgery, Keio University School of Medicine, Tokyo, Japan

**Keywords:** Biochemistry, Cell biology, Biological sciences research methodologies

## Abstract

Human pluripotent stem cell-derived cardiomyocyte (hPSC-CM) differentiation can improve using chemical compounds which mimic early heart development. However, variations in hPSC-CM differentiation efficiency and its poor reproducibility have remained a challenge. Here, we report a unique metabolic method to promote hPSC-CM differentiation that involves marked suppression of the mitochondrial oxidative phosphorylation from the mesendoderm to the cardiac mesoderm, which is regulated by PHGDH, a rate-limiting enzyme in the serine synthesis pathway. Mechanistically, PHGDH inhibition impairs mitochondrial respiration by blocking the electron transport chain, resulting in elevated ROS levels and promoting the cardiomyocyte lineage specification by disrupting the cardiopharyngeal mesoderm lineage differentiation. Additionally, antioxidant supplementation can scavenge ROS and eliminate the effects of PHGDH inhibition. Collectively, our findings show that serine synthesis pathway can regulate cardiomyocyte lineage specification and have implications in providing a cellular source for transplantation and elucidating the potential mechanisms of heart development and pathogenesis of heart disease.

## Highlights

• Energy metabolism changes dramatically during cardiomyocyte differentiation
• PHGDH inhibition promotes cardiomyocyte differentiation
• PHGDH inhibition disrupts cardiopharyngeal mesoderm differentiation
• PHGDH inhibition produces ROS via impairing complex I function

## Graphical abstract

**Figure:**
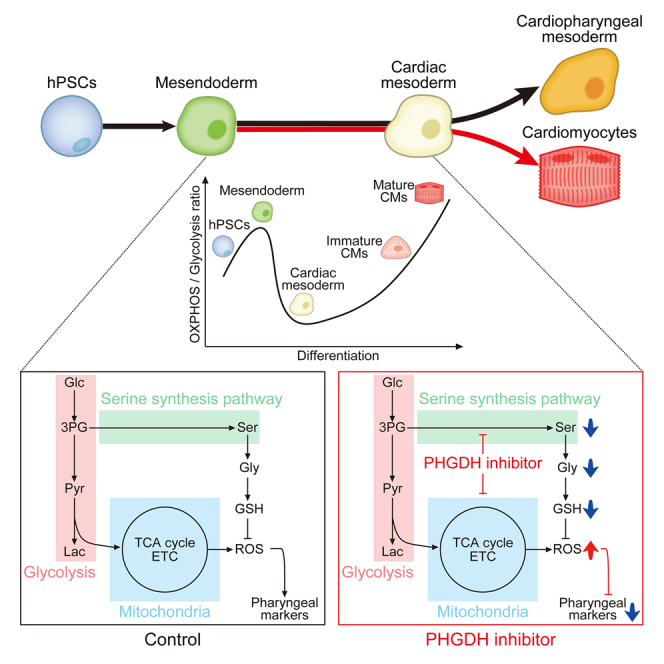

## Introduction

Previous research has mainly focused on elucidating the roles of cytokines and growth factors in heart development.^1^^,^^2^ The differentiation of human pluripotent stem cell (hPSC)-derived cardiomyocytes (hPSC-CMs) has been improved by the use of chemical compounds, including WNT, activin, and bone morphogenic protein signaling modulators, which mimic early heart development.^3^^,^^4^ Notably, monolayer-directed differentiation combined with the sequential activation and inhibition of WNT signaling enhances the generation of hPSC-CMs.^3^ Nevertheless, variations in differentiation efficiency and poor reproducibility have remained challenges, attributing to reasons such as initial seeding densities and confluency and the effect of batch variability of chemical compounds.^5^^,^^6^ Moreover, the mammalian heart contains a distinct population of CMs, smooth muscles, endothelial cells, and cardiac fibroblasts, which are derived from multipotent cardiac progenitors.^7^ Chemical compounds used for CM differentiation have also been applied to induce other mesoderm and endoderm lineages such as skeletal muscles and hepatocytes.^8^^,^^9^ This highlights the process complexity due to the spatiotemporal regulation of signaling networks in organ development.

It is only in the past decade that nutrient resources modulating metabolic pathways have emerged as key regulators of stem cell differentiation.^10^^,^^11^^,^^12^^,^^13^^,^^14^ Erythroid specification of hematopoietic stem cells requires glutamine-dependent nucleotide synthesis, and blocking this pathway diverts into myelomonocytic lineages even under erythropoietin treatment.^12^ In skeletal stem cells, α-ketoglutarate (αKG) derived from glutamine is critical for stem cell proliferation and osteoblast specification, but not for differentiation to adipocyte lineages.^13^ Interestingly, in hPSCs, specification into three germ layers has unique lineage-specific glutamine metabolism.^14^ These studies support that nutrient metabolism not only maintains bioenergetic homeostasis but also directly regulates stem cell fate. Therefore, metabolism is a critical hallmark of stem cell biology.^15^

hPSCs, including human embryonic stem cells (hESCs) and human-induced pluripotent stem cells (hiPSCs), generate energy mainly through glycolysis.^16^ During the exit from pluripotency and initial differentiation into mesoderm and endoderm lineages, energy production shifts from glycolysis to mitochondrial oxidative phosphorylation (OXPHOS).^17^ This metabolic switch is thought to be required to cover the energy demands of the different cell types. However, during early ectoderm specification, high glycolytic flux is maintained via elevated MYC activity.^18^ Recent studies have reported that activating mitochondrial OXPHOS using exogenous pyruvate or glutamine oxidation in the tricarboxylic acid cycle promotes mesoderm differentiation.^14^^,^^19^ However, little is known about how energy metabolism changes over time during cardiac differentiation and how it may contribute to CM lineage commitment, which belongs to the mesoderm.

In this study, we investigated the energy demand for CM differentiation of hPSCs. Surprisingly, we found that mitochondrial OXPHOS was markedly suppressed in the transition from the mesendoderm to the cardiac mesoderm. In hPSCs, phosphoglycerate dehydrogenase (PHGDH), a rate-limiting enzyme in the serine synthesis pathway (SSP), regulates mitochondrial function, and the inhibition of PHGDH impairs mitochondrial respiration. SSP also attenuates oxidative stress by supporting the production of reduced glutathione (GSH).^20^ Additionally, we found that inhibition of PHGDH using small molecules in the mesendoderm promoted CM lineage specification by suppressing cardiopharyngeal mesoderm (CPM) lineages. The generation of reactive oxygen species (ROS) due to the impairment of mitochondrial respiration regulates CMs and CPM lineage specification. Thus, our study suggests that the energy metabolism can coordinate and facilitate the determination of stem cell fate.

## Results

### Inhibition of SSP promotes CM differentiation

**Figure 1:**
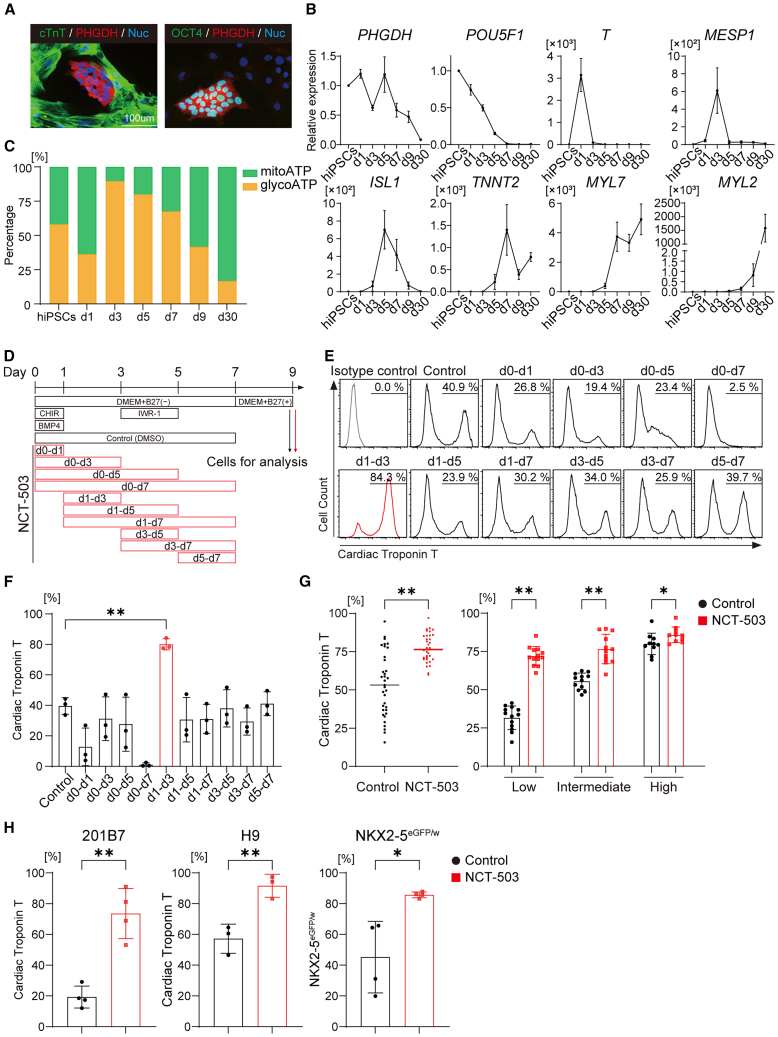
Inhibition of SSP promotes CM differentiation (A) Representative image of immunocytochemistry of co-cultured hiPSCs and hiPSC-CMs. (B) The chronological expression of *PHGDH* and differentiation stage-specific genes were assessed using qRT-PCR analysis. mRNA expression of hiPSCs was defined as the control value. Student’s *t* test was performed for each gene (*n* = 3). (C) The mitochondrial and glycolytic ATP production during hiPSC-CM differentiation was measured using a flux analyzer. (D) Schematic illustration of the hPSC-CM differentiation protocol and timing of NCT-503 treatment. (E) Representative flow cytometry analysis for cardiac Troponin T expression on Day 9 with the timing of each NCT-503 addition. (F) Proportion of cardiac Troponin T-positive cells under each condition. One-way analysis of variance (ANOVA) with Dunnett’s test was performed (*n* = 3). (G) The left scatterplot displays the proportion of cardiac Troponin T-positive cells on Day 9 in 253G4 with control or NCT-503 treatment from Day 1 to Day 3. Student’s *t* test was performed (*n* = 35). The right bar graph displays the induction efficiency in control divided into three groups: High (>70%, *n* = 13), Intermediate (45%–70%, *n* = 12), and Low (<45%, *n* = 10). Student’s *t* test was performed for each group. (H) Proportion of cardiac Troponin T-positive cells on Day 9 in each hPSC line (201B7, H9, and NKX2-5^eGFP/w^) with control or NCT-503 treatment from Day 1 to Day 3. Student’s *t* test was performed (*n* = 4). ∗*p* < 0.05; ∗∗*p* < 0.01. Data are shown as mean ± SD. Experimental repeats were completely independent. ATP, adenosine triphosphate; BMP4, bone morphogenetic protein 4; cTnT, cardiac Troponin T; DMEM, Dulbeccoʼs Modified Eagleʼs Medium; DMSO, dimethyl sulfoxide; hiPSCs, human induced pluripotent stem cells.

To identify metabolic features of hPSCs and hPSC-derived cells, we performed RNA sequencing (RNA-seq) analysis with a focus on metabolism-related enzymes in hPSCs and hPSC-CMs (as terminally differentiated cells purified by means of metabolic selection).^21^^,^^22^ The RNA-seq analysis revealed that all four hPSC lines (253G4, 201B7, H9, and NKX2-5^eGFP/w^) exhibited >10-fold higher expression of the *de novo* SSP enzymes, *PHGDH* and *PSAT1*, than hPSC-CMs (Figures S1A and S1B), in line with previous reports.^21^^,^^23^ The SSP branches from glycolysis and is involved in numerous metabolic pathways, including nucleotide or GSH synthesis.^20^ This high expression of the SSP genes suggested that hPSCs are actively proliferating. We confirmed the difference in PHGDH expression between hPSCs and hPSC-CMs using immunocytochemistry and western blotting (Figures 1A and S1C). Therefore, we focused on the functions of SSP during CM differentiation.

Next, we evaluated changes in *PHGDH* expression during CM differentiation from hiPSCs to hiPSC-derived CMs (hiPSC-CMs). Interestingly, *PHGDH* expression markedly decreased from the mesendoderm (Day 1) to the cardiac mesoderm (Day 3) (Figure 1B). The SSP functions to maintain mitochondrial respiration and homeostasis.^24^ To identify the unique energy metabolic changes during CM differentiation, we evaluated the chronological changes in the energy balance between glycolysis and mitochondrial OXPHOS from hiPSCs to hiPSC-CMs using a flux analyzer (Figure 1C). Real-time ATP rate assays revealed that mitochondrial OXPHOS decreased markedly in the cardiac mesoderm (Day 3), consistent with the timing of the decrease in *PHGDH* expression (Figures 1B and 1C).

To evaluate the importance of the SSP during CM differentiation, we applied a small molecule inhibitor targeting PHGDH (NCT-503) to the CM differentiation protocol (Figure 1D). Flow cytometric analysis of cardiac Troponin T revealed that hiPSCs could be differentiated into CMs stably and efficiently with NCT-503 treatment from Day 1 to Day 3 (Figures 1E and 1F; Videos S1 and S2). We observed variation under the same culture conditions in the control group (Figure 1G). When we divided the induction efficiency in the control group into three groups —High (>70%), Intermediate (45%–70%), and Low (<45%)— NCT-503 treatment improved CM differentiation efficiency in all of the groups (Figure 1G). Therefore, to clarify differences due to interventions, we used a CM differentiation efficiency of ∼40% for the control group. Interestingly, the total cell numbers did not differ under PHGDH inhibition, and the absolute number of harvested CMs increased 2-fold (Figure S1D). We confirmed stable CM differentiation efficiency and good reproducibility in all four hPSC lines (Figure 1H).

Video S1. Spontaneous Beating hiPSC-CMs under Control Condition on Day 9, related to Figure 1

Video S2. Spontaneous Beating hiPSC-CMs under NCT-503 Treatment Condition on Day 9, related to Figure 1

**Figure 2:**
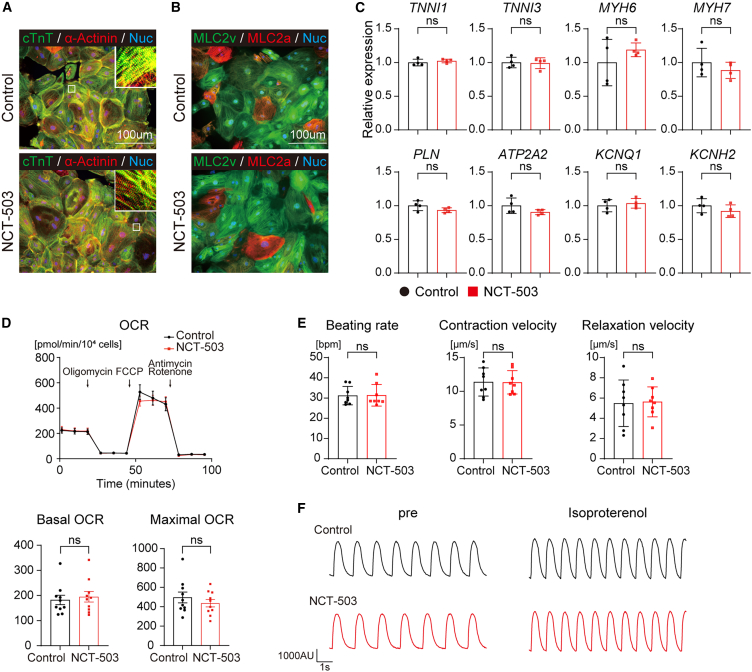
hiPSC-CMs induced with SSP inhibition have no significant effects on morphological and functional characteristics (A) Representative image of immunocytochemistry for α-actinin (red) and cardiac Troponin T (green) in control or NCT-503 hiPSC-CMs on Day 30. Cell nuclei are stained with Hoechst (blue). The high-magnification view in the inset shows the sarcomeric organization. (B) Representative image of immunocytochemistry for MLC2a (red) and MLC2v (green) in control or NCT-503 hiPSC-CMs on Day 30. Cell nuclei are stained with Hoechst (blue). (C) qRT-PCR analysis of cardiac isoforms, calcium handling-related, and potassium ion channel genes in control or NCT-503 hiPSC-CMs on Day 30. The mRNA expression of hiPSC-CMs in the control group was defined as the control value. Student’s *t* test was performed (*n* = 4). (D) Representative mitochondrial OCR of control or NCT-503 hiPSC-CMs on Day 30 was measured using a flux analyzer. We sequentially applied oligomycin (1.5 μg/mL), FCCP (0.5 μM), Antimycin A (1 μM), and rotenone (1 μM). Quantification of the basal and maximal OCRs in each group. Student’s *t* test was performed (*n* = 10). (E) Contractile functions measured using video imaging with motion vector in control and NCT-503 hiPSC-CMs on Day 30. Student’s *t* test was performed (*n* = 8). (F) Responses to isoproterenol (1000 nM) of control or NCT-503 hiPSC-CMs on Day 30. Scale bar: 100 μm. Data are shown as mean ± SD (C and E) or mean ± SEM (D). Experimental repeats were completely independent (C and E) or simultaneous well-replicates (D). cTnT, cardiac Troponin T; OCR, oxygen consumption rate.

Next, we evaluated the differences in hiPSC-CMs induced using NCT-503 or the vehicle control [dimethyl sulfoxide (DMSO)], on Day 30. Both hiPSC-CMs showed striated muscle structure in Troponin T and α-actinin staining, with no significant difference between them (Figure 2A). Most hiPSC-CMs exhibited MLC2v-positive ventricular CMs, whereas some showed MLC2a-positive immature or atrial CMs (Figure 2B). There were no significant differences in the expression of cardiac isoforms of troponin I (*TNNI1* and *TNNI3*), myosin heavy chain (*MYH6* and *MYH7*), calcium handling-related genes (*PLN* and *ATP2A2*), or potassium ion channels (*KCNQ1* and *KCNH2*) (Figure 2C). Furthermore, MitoStress test analysis revealed no significant differences in the oxygen consumption rate (OCR), an indicator of mitochondrial respiration, in both hiPSC-CMs (Figure 2D). Beating rate, contraction velocity, and relaxation velocity showed no significant difference between the groups (Figure 2E). In the drug response analysis using FDSS/μCELL, both hiPSC-CMs had a good response, with isoproterenol showing no significant differences (Figure 2F). Taken together, these findings suggested that PHGDH inhibition from Day 1 to Day 3 can promote CM differentiation, with no significant effect on the morphological and functional characteristics.

### Inhibition of SSP promotes CM specification by disrupting CPM differentiation

**Figure 3:**
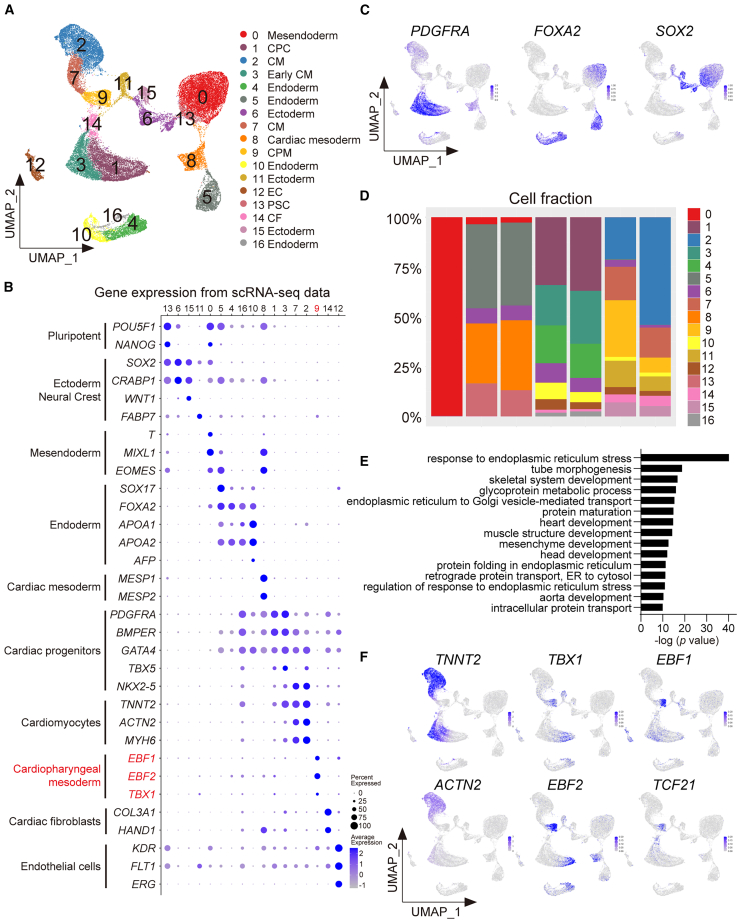
scRNA-seq analysis reveals changes in cell populations under SSP inhibition (A) The UMAP plot of scRNA-seq data displays individual cells by cell types. (B) The dot plot shows key marker genes identified in each cell cluster. (C) UMAP plot showing the expression of genes indicative of mesoderm, endoderm, and ectoderm. (D) Bar plot showing the distribution of cells in each sample. (E) Bar graph showing GO enrichment analysis for the upregulated genes in Cluster 9. (F) UMAP plot showing the expression of genes indicative of CM (*TNNT2* and *ACTN2*) and CPM (*TBX1*, *EBF1*, *EBF2*, and *TCF21*). CF, cardiac fibroblast; CM, cardiomyocyte; CPC, cardiac progenitor cell; CPM, cardiopharyngeal mesoderm; EC, endothelial cell; ER, endoplasmic reticulum; PSC, pluripotent stem cell; scRNA-seq, single-cell RNA sequencing; UMAP, Uniform Manifold Approximation and Projection.

To better characterize the signatures of cell populations induced by PHGDH inhibition, we interrogated the transcriptomes of both control and NCT-503-treated samples using single-cell RNA sequencing (scRNA-seq) analysis (Figure S2A). Unsupervised clustering of a total of 42881 individual cells, including 7749 cells from Day 1 (d1), 3257 cells from the Day 3 control group (d3D), 2788 cells from the Day 3 NCT-503-treated group (d3N), 7929 cells from the Day 5 control treated group (d5D), 8098 cells from the Day 5 NCT-503-treated group (d5N), 4237 cells from the Day 9 control treated group (d9D), and 8823 cells from the Day 9 NCT-503-treated group (d9N), identified 17 clusters (Figures 3A and S2B). We found differentially expressed genes (DEGs) in each cluster and ascribed cell identity using Gene Ontology (GO) enrichment analysis along with well-established marker genes (Figures 3B and S2C and S2D). Day 1 was represented by the mesendoderm (Cluster 0), which expressed *T*, *MIXIL1*, and *EOMES*. Day 3 was represented by the cardiac mesoderm (Cluster 8), which expressed *MESP1* and *MESP2*; definitive endoderm (Cluster 5), which expressed *SOX17* and *FOXA2*; and ectoderm (Cluster 6), which expressed *SOX2* and *CRABP1*. Day 5 was represented by cardiac progenitors (Cluster 1), which expressed *PDGFRA*, *BMPER*, and *GATA4*; early CM (Cluster 3), which expressed *TNNT2*; the endoderm (Clusters 4, 10, and 16), which expressed *APOA1*, *APOA2*, and *AFP*; and the ectoderm (Cluster 6). Day 9 was represented by CM (Cluster 2), which expressed *TNNT2*, *ACTN2*, and *MYH6*; cardiac progenitors (Cluster 7), which expressed *GATA4* and *NKX2-5*; CPM (Cluster 9), which expressed *EBF1*, *EBF2*, and *TBX1*; endothelial cells (Cluster 12), which expressed *KDR*, *FLT1*, and *ERG*; cardiac fibroblasts (Cluster 14), which expressed *COL3A1* and *HAND1*; and endoderm (Cluster 10), ectoderm (Cluster 11), and neural crest (Cluster 15), which expressed *WNT1* and *FABP7*. We identified three germ layer cell populations in our scRNA-seq datasets (Figure 3C). Consistent with the results of the flow cytometry analysis (Figure 1G), the CM cluster (Cluster 2) was more prevalent in d9N than in d9D (Figure 3D). For precise analysis, we classified the TNNT2-positive CM populations into eight subclusters (Figure S2E). The clustering pattern of the CMs was primarily determined based on developmental stage (Figure S2F). There was no notable difference in the distribution of CM clusters between the control and NCT-503-treated groups (Figure S2E). There were fewer populations expressing *NR2F2* or *SHOX2*, which were markers of atrial- or sinoatrial node-type CMs, respectively (Figure S2F). These data and our immunocytochemistry of MLC2v on Day 30 (Figure 2B) suggest that most CMs were of the ventricular-type.

Next, we focused on cells whose differentiation fate changed from non-CMs to CMs upon the inhibition of PHGDH. The expression pattern of stage-specific genes other than PHGDH showed similar trends among the two groups in our scRNA-seq datasets (Figure S2G). As the demand for SSP may increase after PHGDH inhibition, the expression of PHGDH was higher in the NCT-503-treated group compared with that in control group. Interestingly, one-quarter of the cells in d9D contained a Cluster 9 population, whereas it was much less abundant in the cells in d9N (Figure 3D). GO enrichment analysis of DEGs in Cluster 9 showed that genes were involved in CPM development such as skeletal system and head development (Figure 3E). Markers for CPM (*TBX1*, *EBF1*, *EBF2*, and *TCF21*) were highly expressed in Cluster 9, but markers for CM (*TNNT2* and *ACTN2*) were less highly expressed (Figure 3F). CPM gives rise to several lineages, including skeletal muscles in the head and neck, as well as second heart field-derived cardiovascular structures.^25^ The *Tbx1* gene, encoding a T-box transcription factor, is expressed in the CPM, which is derived from the second heart field.^26^^,^^27^^,^^28^ *TBX1* is the major gene for the 22q11.2 deletion syndrome known as DiGeorge syndrome, which is characterized by defects in the developmental structures derived from the CPM.^29^ There were no notable differences in the distribution of clusters other than those of CM and CPM on Day 9 (Figure 3D).

**Figure 4:**
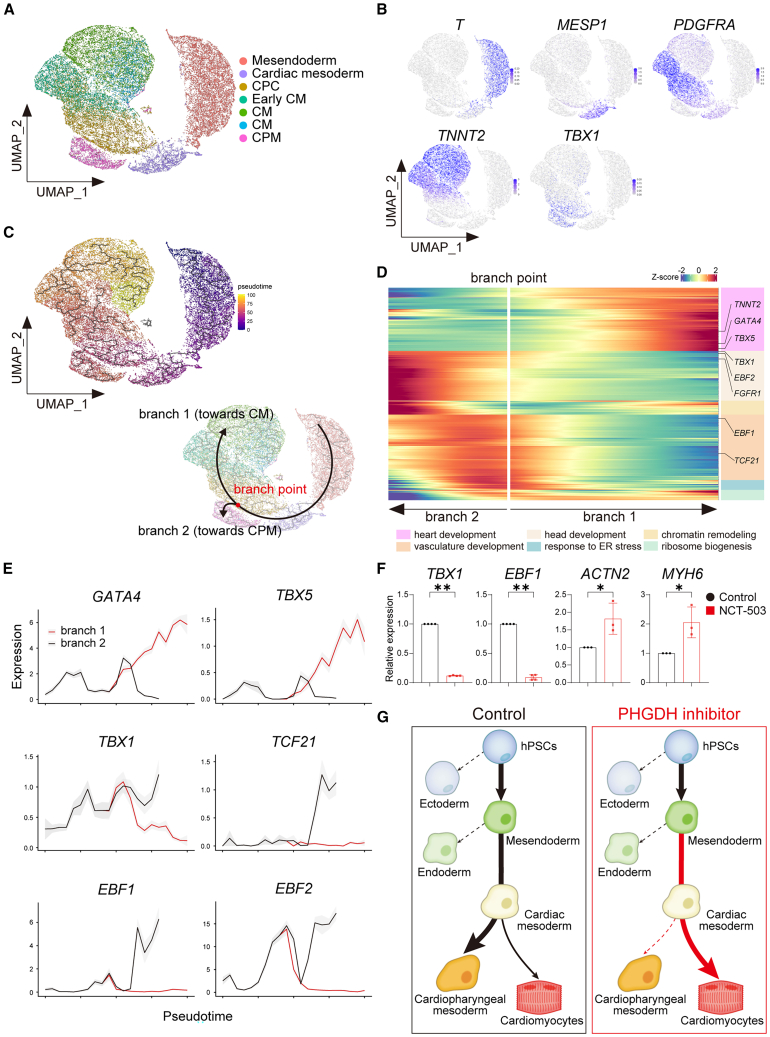
Inhibition of SSP promotes CM specification by disrupting CPM differentiation (A) UMAP plot of scRNA-seq data displaying individual cells by cell type. Endoderm and ectoderm cells were excluded from this analysis. (B) UMAP plot showing the expression of genes indicative of the mesendoderm (*T*), cardiac mesoderm (*MESP1*), cardiac progenitor (*PDGFRA*), CM (*TNNT2*), and CPM (*TBX1*). (C) UMAP plot showing the pseudotime of cardiac differentiation with inferred trajectories. Potential differentiation trajectories are schematically depicted with arrows. (D) Heatmap illustrating genes linked to cell fate divergence at branch points. (E) Line graph showing the expression of key genes that diverged at the branchpoint. (F) The graph shows qPCR analysis of CPM and CM markers on Day 9 with control or NCT-503 treatment from Day 1 to Day 3. mRNA expression under the control condition was defined as the control value. Student’s *t* test was performed (*n* = 3). (G) Schematic illustration of hPSC-CM differentiation under control or PHGDH inhibition. ∗*p* < 0.05; ∗∗*p* < 0.01. Data are shown as mean ± SD. Experimental repeats were completely independent. CM, cardiomyocyte; CPC, cardiac progenitor cell; CPM, cardiopharyngeal mesoderm; ER, endoplasmic reticulum; scRNA-seq, single-cell RNA sequencing; UMAP, Uniform Manifold Approximation and Projection.

To identify cells whose differentiation fate changed from CPM to CM upon PHGDH inhibition, we employed pseudotime analysis to construct the differentiation trajectory with our scRNA-seq data (Figure 4A). To focus on CM and CPM differentiation, ectoderm and endoderm cells were excluded from this analysis. Cells were successfully deployed at the developmental stage (Figure 4B). The trajectory depicted successful bifurcation into two branches, representing a branch to CM (branch 1) and a branch to CPM (branch 2) (Figure 4C). *T* and *MESP1* were highly expressed in the pre branch, *PDGFRA* was expressed in cells both before and after branching, *TNNT2* was highly expressed in the population toward branch 1, and *TBX1* was highly expressed in the population toward branch 2 (Figure 4B).

To comprehensively view the differentiation trajectory, we analyzed 1187 DEGs and observed 6 gene expression clusters with different patterns (Figure 4D). Genes in the cluster enriched with heart development genes were gradually upregulated toward branch 1 (Figure 4D). Conversely, genes in the cluster enriched with pharyngeal development genes, such as those involved in head and vasculature development, were progressively downregulated toward branch 1 (Figure 4D). For example, *GATA4* and *TBX5* were enriched in cells that followed the CM trajectory, whereas *TBX1*, *TCF21*, *EBF1*, and *EBF2* were prevalent in cells within the CPM branch (Figure 4E). Consistent with our scRNA-seq analysis, the expressions of *TBX1* and *EBF1* were significantly lower in NCT-503-treated cells, whereas that of *ACTN2* and *MYH6* was higher than that in control cells (Figures 4F and S3A). In summary, these data suggest that PHGDH inhibition from Day 1 to Day 3 promoted CM differentiation, instead of CPM differentiation (Figure 4G).

### SSP regulates mitochondrial energy metabolism

**Figure 5:**
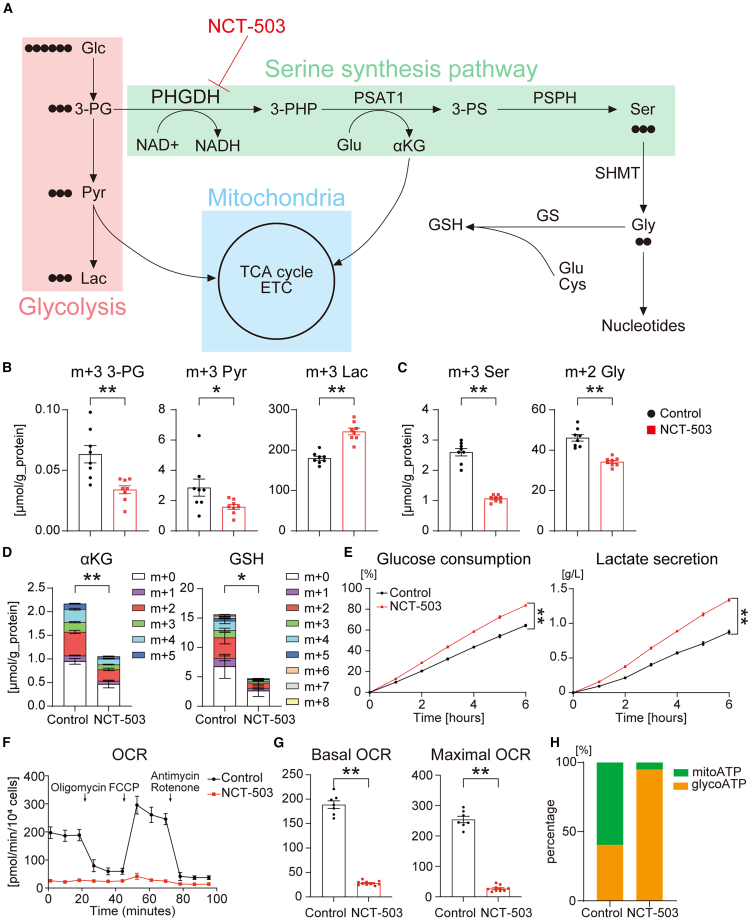
SSP regulates mitochondrial energy metabolism (A) A schematic of the metabolic map of [U-^13^C]-labeled glucose metabolism related to SSP. (B) ^13^C-labeled metabolites related to glycolysis, under control or NCT-503 treatment condition (*n* = 8). (C) ^13^C-labeled serine and glycine under each condition (*n* = 8). (D) The distributions of α-ketoglutarate and glutathione isotopomers under each condition (*n* = 8). (E) Concentrations of glucose consumption and lactate secretion under each condition (*n* = 6). (F) Representative mitochondrial OCR under each condition, performed on differentiation Day 1 with control or NCT-503 treatment, measured using a flux analyzer (*n* = 10). We sequentially applied oligomycin (0.5 μg/mL), FCCP (0.25 μM), Antimycin A (1 μM), and rotenone (1 μM). (G) Quantification of basal and maximal OCRs under each treatment condition (*n* = 10). (H) Graph showing the ratio of mitochondrial and glycolytic ATP production, performed on differentiation Day 1 with control or NCT-503 treatment, measured using a flux analyzer (*n* = 10). Student’s *t* test was performed for each analysis. ∗*p* < 0.05; ∗∗*p* < 0.01. Data are shown as mean ± SEM. Experimental repeats were completely independent (B, C, D, and E) or simultaneous well-replicates (F, G, and H). 3-PHP, 3-phosphohydroxypyruvate; 3-PG, 3-Phosphoglycerate; 3-PS, 3-phosphoserine; αKG, α-ketoglutarate; ATP, adenosine triphosphate; Cys, cysteine; ETC, electron transport chain; Glc, glucose; Glu, glutamate; Gly, glycine; GSH, glutathione; Lac, lactate; OCR, oxygen consumption rate; PHGDH, phosphoglycerate dehydrogenase; PSAT1, phosphoserine aminotransferase 1; Pyr, pyruvate; PHPH, phosphoserine phosphatas; Ser, serine; SHMT, serine hydroxymethyltransferase; SSP, serine synthesis pathway; TCA cycle, tricarboxylic acid cycle.

To investigate the function of SSP during CM differentiation, we performed capillary electrophoresis/mass spectrometry-based metabolome analysis with [U-^13^C]-labeled glucose and NCT-503 on Day 1 (Figure 5A). The samples were harvested 12 h after changing the culture conditions. The ^13^C-labeled glucose-derived glycolytic metabolites, 3-phosphoglycerate and pyruvate, decreased in the NCT-503-treated cells, with an increase in glucose incorporation into lactate (Figure 5B). Glucose entry into the SSP was abrogated upon PHGDH inhibition, with decreased ^13^C labeling of serine and glycine, verifying the targeting effect of NCT-503 (Figures 5A and 5C). There was a decrease in the tricarboxylic acid metabolite αKG, which is synthesized from glutamate via PSAT1, in the NCT-503-treated cells (Figure 5D). There was a decrease in the antioxidant GSH, which is synthesized from glutamate, cysteine, and glycine, in the NCT-503-treated cells, consistent with the decrease in ^13^C-labeled glycine (Figure 5D). Unexpectedly, purine nucleotides were maintained in the NCT-503-treated cells (Figure S4A). Upon independent measurement, the glucose consumption and extracellular lactate secretion levels were also found to be elevated in the NCT-503-treated cells compared to control (Figure 5E). These data suggested that NCT-503 inhibited SSP and accelerated glycolytic metabolism.

To confirm the impact of PHGDH inhibition on mitochondrial energy metabolism, we used a flux analyzer to evaluate the OCR (Figure 5F). Basal and maximal OCRs were significantly lower in the NCT-503-treated group than those in the control group (Figure 5G). Next, we investigated the effects of PHGDH inhibition on the energy balance between glycolysis and mitochondrial OXPHOS using a flux analyzer. Real-time ATP rate assay revealed that mitochondrial OXPHOS decreased markedly upon PHGDH inhibition (Figure 5H). To investigate the relationship between energy metabolism and SSP function more precisely, PHGDH expression was knocked down using a small interfering RNA (siRNA) (Figure S4B), following which the OCR was evaluated using a flux analyzer. The PHGDH knocked-down cells exhibited a lower OCR than those transfected with the non-targeting siRNA (Figure S4C). Furthermore, we confirmed no significant changes in cell cycle assay and apoptosis assay (Figures S4D and S4E). Taken together, these data indicate that PHGDH regulates mitochondrial energy metabolism and that inhibition of PHGDH, which promotes CM differentiation; this is not due to a selection.

### Mitochondrial stress-induced ROS inhibit CPM differentiation

Since SSP is active in hPSCs, we investigated the effect of PHGDH inhibition on undifferentiated state. Immunohistochemistry for OCT4 and NANOG showed an undifferentiated state in the NCT-503-treated cells (Figure S5A). Previous studies have reported that SSP coordinates the nucleotide levels by maintaining the central carbon metabolism in cancer cells that express high PHGDH.^30^ NCT-503 suppressed hiPSC proliferation and supplementation of nucleosides completely rescued the same (Figure S5B). However, from Day 1 to Day 3, nucleoside supplementation did not cancel out the effect of PHGDH inhibition on CM differentiation (Figures S5C and S5D). This is consistent with the results of the metabolome analysis carried out in this study, which showed no decrease in the nucleotide levels (Figure S4A). These results indicated that PHGDH inhibition, which promotes CM differentiation, is not due to loss of undifferentiated markers or cell proliferation.

**Figure 6:**
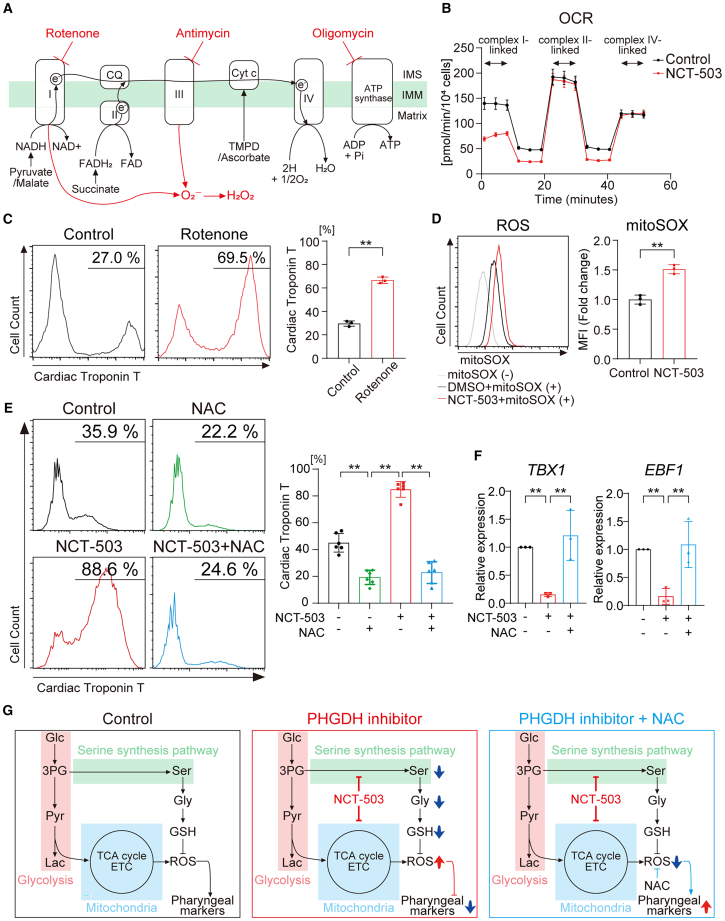
Mitochondrial stress-induced ROS inhibits CPM differentiation (A) Schematic of the key steps in mitochondrial ETC. (B) Representative mitochondrial respiratory chain assay using XF PMP, performed on differentiation Day 1 with control or NCT-503 treatment, measured using a flux analyzer (*n* = 4). (C) Representative flow cytometry analysis for cardiac Troponin T expression on Day 9, with control or rotenone (5 nM) treatment from Day 1 to Day 3. The graph displays the proportion of cardiac Troponin T-positive cells under each condition. Student’s *t* test was performed for each analysis (*n* = 3). (D) Representative flow cytometry analysis for reactive oxygen species, performed on differentiation Day 1 using MitoSOX, under control (black) or NCT-503 (red) treatment condition. The gray line indicates only cells without MitoSOX. The graph displays the fold-change in mean fluorescence intensity for MitoSOX expression, relative to that under control conditions. Student’s *t* test was performed for each analysis (*n* = 3). (E) Representative flow cytometry analysis for cardiac Troponin T expression on Day 9, with control, NAC (10 mM), NCT-503, or NCT-503+NAC treatment from Day 1 to Day 3. The graph displays the proportion of cardiac Troponin T-positive cells under each condition. One-way ANOVA with Dunnett’s test was performed (*n* = 6). (F) qPCR analysis of CPM markers on Day 9 with each treatment from Day 1 to Day 3. The mRNA expression under the control condition was taken as the control value. One-way ANOVA with Dunnett’s test was performed (*n* = 3). (G) Schematic of the metabolic features under control, NCT-503, or NCT-503+NAC treatment condition. ∗*p* < 0.05; ∗∗*p* < 0.01. Data are shown as mean ± SD (C, D, E, and F) or mean ± SEM (B). Experimental repeats were completely independent (C, D, E, and F) or simultaneous well-replicates (B). 3-PG, 3-Phosphoglycerate; ADP, adenosine diphosphate; ATP, adenosine triphosphate; CQ, coenzyme Q; Cyt *c*, cytochrome *c*; DMSO, dimethyl sulfoxide; ETC, electron transport chain; FADH2, flavin adenine dinucleotide; Glc, glucose; Gly, glycine; GSH, glutathione; IMM, inner mitochondrial membrane; IMS, intermembrane space; Lac, lactate; MFI, mean fluorescence intensity; NAC, *N*-acetyl-L-cysteine; NADH, nicotinamide adenine dinucleotide; OCR, oxygen consumption rate; Pyr, pyruvate; ROS, reactive oxygen species; Ser, serine; SSP, serine synthesis pathway; TCA cycle, tricarboxylic acid cycle.

Next, we investigated the effects of SSP on mitochondrial respiration. The Agilent Seahorse XF Cell Mito Stress Test shows a general mitochondrial bioenergetic profile (Figures 5F and 5G). However, many oxidizable substrates are unable to cross the plasma membrane freely, preventing control over which substrates the mitochondria are oxidizing. Mitochondrial respiratory chain assay using XF Plasma Membrane Permeabilizer (XF PMP) allows experimental control over the specific substrates offered to *in situ* mitochondria. Therefore, mitochondrial respiratory chain assay offers a powerful approach to measure mitochondrial electron transport chain (ETC) complex activity in permeabilized cells.^31^ Mitochondrial OXPHOS relies on an ETC consisting of four main enzyme complexes (I–IV) and two mobile electron carriers (coenzyme Q and cytochrome c) (Figure 6A). In this method, permeabilized cells initially receive complex I-linked substrates, pyruvate and malate. Rotenone inhibits complex I and halts NADH-linked respiration. Next, succinate drives respiration from electrons fed directly into the ubiquinone pool via succinate dehydrogenase (complex II) and bypassing complex I inhibition. The addition of Antimycin A inhibits complex III and abolishes this rate. Lastly, the injection of ascorbate with *N*,*N*,*N*′,*N*′-tetramethyl-*para*-phenylene-diamine (TMPD) bypasses the block at complex III and delivers electrons directly to cytochrome *c* oxidase (complex IV). Mitochondrial respiratory chain assay revealed that NCT-503 impaired complex I-linked respiration, while the capacity for complex II- and IV-linked respiration remained intact (Figure 6B). To confirm the inhibitory effect of NCT-503 on complex I, we applied the mitochondrial complex I-specific inhibitor rotenone to induce CM differentiation. Interestingly, hiPSCs could be differentiated into CMs stably and efficiently using rotenone from Day 1 to Day 3, consistent with the results obtained using NCT-503 (Figures 1E, 1F, and 6C).

Mitochondria are the main source of cellular ROS. Mitochondrial complex I and complex III, especially complex I, are considered to be the main sites of ROS production.^32^ ROS can be generated during the transfer of electrons from NADH to coenzyme Q in complex I. Rotenone inhibits the electron transfer from complex I to coenzyme Q. The subsequent leakage of electrons from complex I leads to partial reduction of oxygen to form superoxide ion (O_2_^−^), which is the precursor for ROS. O_2_^−^ is dismutated to hydrogen peroxide (Figure 6A). Furthermore, acute hypoxia produces a superoxide burst during the first few minutes that complex I participates in this process.^33^ On the other hand, oligomycin, a specific inhibitor of ETC complex V (ATP synthase), has a mild effect on ROS generation.^34^ We confirmed that the addition of rotenone or Antimycin A, a specific inhibitor of ETC complex III, promoted CM differentiation, but oligomycin did not (Figures 6C and S5E, S5F). Next, we investigated the ROS production using the mitochondrial superoxide indicator, MitoSOX. MitoSOX-based flow cytometry revealed that NCT-503 treatment enhanced mitochondrial ROS generation (Figure 6D). The antioxidant *N*-acetyl-L-cysteine (NAC) can scavenge mitochondrial ROS and maintain GSH levels.^35^ Supplementation of NAC from Day 1 to Day 3 inhibited CM differentiation and was sufficient to cancel out the PHGDH inhibition (Figure 6E). Furthermore, there was a significant increase in the expression of the CPM-related genes upon NAC supplementation (Figure 6F). These data confirmed that the increase in mitochondrial ROS induced by PHGDH inhibition promoted CM differentiation, to prevent hiPSCs from differentiating into the CPM lineage.

Finally, we investigated what classical pathways were affected by PHGDH inhibition. CM differentiation efficiency was evaluated by adding WNT, TGFβ/SMAD, and FGF/ERK pathway inhibitors under NCT-503 plus NAC condition. Interestingly, the inhibition of the FGF/ERK pathway using PD173074 can promote CM differentiation even under NCT-503 plus NAC conditions (Figures S5G and S5H). The canonical FGF/ERK pathway is necessary and sufficient to promote pharyngeal muscle specification in the cardiopharyngeal lineage.^36^^,^^37^ Our results indicate that SSP may regulate cardiac differentiation via modulating the classical FGF/ERK pathway.

In summary, SSP regulated the energy balance between glycolysis and OXPHOS during CM differentiation. In mesendodermal cells, SSP maintains the antioxidant GSH and mitochondrial OXPHOS. Inhibition of PHGDH generates mitochondrial ROS via the impairment of complex I function and prevents CPM lineage differentiation. Under PHGDH inhibition, NAC supplementation reduced mitochondrial ROS and induced CPM lineage differentiation (Figure 6G).

## Discussion

Heart disease is the leading cause of death worldwide. Although treatments for heart failure have advanced greatly, heart transplantation remains the only curative treatment for patients with end-stage heart failure. Heart transplantation has become a major challenge because the number of individuals with need for heart transplantation is increasing while the donor supply is limited.^38^ To overcome this challenge, the generation of CMs from hPSCs has been studied, which has great potential for the development of new treatments. hPSC-CM differentiation has been improved with chemical compounds that modulate signaling pathways, but variations in CM differentiation efficiency and poor reproducibility remain challenging. These variations have been ascribed to the initial seeding densities and confluency and the effect of the batch variability of chemical compounds.^5^^,^^6^ In this study, we proposed a unique metabolic approach to overcome these challenges.

Metabolic energy balance is associated with stem cell differentiation.^39^ hPSCs maintain energy metabolism mainly through aerobic glycolysis.^21^ During mesendoderm differentiation, there is a metabolic switch from glycolysis to mitochondrial OXPHOS, as hPSCs exit from the pluripotent state.^17^^,^^40^ hPSC-CMs have more matured mitochondria than hPSCs, and mitochondrial OXPHOS is superior to glycolysis in hPSC-CMs.^21^^,^^22^^,^^41^^,^^42^ Therefore, the energy metabolism during CM differentiation was thought to change gradually from glycolysis to mitochondrial OXPHOS. However, our study revealed that mitochondrial OXPHOS is markedly suppressed from the mesendoderm to the cardiac mesoderm and that mitochondrial respiration is regulated by the SSP. Moreover, our study revealed that suppression of mitochondrial respiration by means of PHGDH inhibition in the mesendoderm promotes CM lineage specification. These data indicate that the metabolic energy balance plays an important role in stem cell fate.

Because the SSP provides precursors for a variety of biosynthetic pathways, a pivotal aspect of the SSP is the conversion of serine to glycine by serine hydroxymethyltransferase. Glycine is a major source of methyl groups for the one-carbon pools required for the biosynthesis of GSH, proteins, purine nucleotides, and DNA/histone methylation.^20^ As has been previously reported, in proliferating cells such as cancer cells, the SSP is required to maintain nucleotide synthesis.^30^^,^^43^ This regulation of nucleotide synthesis is independent of serine utilization, and its mechanism is based on disruptions in mass balance.^30^ Therefore, supplementation of nucleosides (not serine itself) is sufficient to rescue the defects observed upon PHGDH inhibition. SSP is active in hiPSCs and inhibition of PHGDH results in decreased hiPSC proliferation. Similar to previous reports, nucleoside supplementation rescued hiPSC proliferation following PHGDH inhibition in this study as well.^30^ However, in the mesendoderm, our metabolome analysis showed that the inhibition of PHGDH did not affect nucleotide synthesis. Flow cytometry analysis of cardiac Troponin T also showed that nucleoside supplementation did not cancel out the effect of PHGDH inhibition. PHGDH inhibition did not affect the undifferentiated state. These data suggested that nucleotide synthesis and residual hPSCs in the mesendoderm are less relevant to CM differentiation.

Considering the central role of PHGDH, it is possible that regulation of stem cell fate is related to metabolic stress. ROS have been implicated in the mechanisms of CM differentiation from hPSCs, but the exact ROS signaling during CM differentiation is poorly understood, functionally and temporally.^44^ The roles of ROS signaling in stem cell fate are dependent on the intensity of stimuli, cellular context, and metabolic state.^45^ Therefore, methodological optimization has been attempted to robustly enhance the generation of CMs through metabolic modulation. Our study revealed that mitochondrial ROS generation during the transition from the mesendoderm to the cardiac mesoderm promotes CM differentiation by inhibiting the CPM lineage. Furthermore, using recent analytical methods, such as scRNA-seq analysis, we were able to clarify changes in cell fate. Our data also revealed that PHGDH inhibition did not affect cell fate other than that of the mesoderm. Indeed, the total cell numbers were not different under PHGDH inhibition and the absolute number of harvested CMs increased due to the improvement of CM differentiation efficiency. Furthermore, we confirmed that PHGDH inhibition did not affect the cell cycle and induce apoptosis. These data indicated that PHGDH inhibition promotes CM lineage specification by suppressing CPM lineage differentiation and not by eliminating cells.

In recent years, metabolism has re-emerged as an active driver of physiological changes rather than merely a housekeeping process during development. Embryogenesis is guided by dynamic shifts in cellular energy demands and the utilization of anabolic precursors.^46^ Metabolism is a tightly regulated process that can be reprogrammed not only to optimize energy production but also to fulfill changing energetic and growth needs while simultaneously influencing gene regulation. With the expanding understanding of metabolic control over cell fate specification, particularly during gastrulation, metabolic regulation is now recognized as a potential signaling axis in the regulation of embryogenesis.^47^^,^^48^ However, little is known about the relationships between heart development and metabolic regulation. hPSCs hold significant potential for advancing our understanding of heart development. Differentiating hPSCs into cardiac cells allows for modeling early cardiac developmental processes and exploring key signaling pathways. This approach enables detailed analysis of gene regulatory networks and cellular behaviors involved in tissue formation. A major challenge lies in the inability to fully replicate the spatiotemporal regulation present in natural heart development. However, our findings suggest that metabolic regulation, especially SSP, may regulate heart development. Further research is required to understand how metabolism regulates heart development.

Taken together, energy metabolism varies dramatically during CM differentiation and interventions in energy metabolism can regulate stem cell differentiation. This study indicates that stage-specific modulation of metabolism possibly results in the specification of CM lineages, providing not only a cellular source for transplantation, but also potential mechanisms for heart development and the pathogenesis of heart disease.

### Limitations of the study

There are some limitations to the present study that should be considered when interpreting the results. Our study showed that the inhibition of PHGDH from Day 1 to Day 3 highly likely acted as promoting CM specification because there was a bifurcation point during the cardiac progenitor phase based on the trajectory analysis using our scRNA-seq dataset. However, it was difficult to rule out the possibility of promoting mesoderm induction because significant transcriptional differences did not appear until Day 9. This may reflect overlapping gene expression at the early stage of differentiation. Alternatively, there may be a time difference between gene expression and epigenetic modifications induced by metabolic stress. In addition, mitochondrial ROS can modulate the FGF/ERK pathway,^49^ but we did not clarify the direct relationships in our model. Metabolism can affect signaling pathways by regulating the activity of proteins through post-translational modifications, but further research is needed to clarify the mechanism.

## Resource availability

### Lead contact

Further information and requests for resources and reagents should be directed to and fulfilled by the lead contact, Shugo Tohyama (shugo.tohyama@fujita-hu.ac.jp).

### Materials availability

This study did not generate new unique reagents.

### Data and code availability

• The RNA-seq and scRNA-seq data have been deposited in the Gene Expression Omnibus and are publicly available as of the date of publication. Accession numbers are listed in the key resources table.
• This paper does not report the original code.
• Any additional information required to reanalyze the data reported in this paper is available from the lead contact upon request.

## Acknowledgments

The authors thank Yuki Yamamoto, Sayaka Kanaami, Kuniko Momoi, Tomoko Haruna, Yui Narita, and Rei Ohno for their technical assistance with cell preparation and culture (Fujita Medical Innovation Center Tokyo, Fujita Health University); Satoru Okamoto for producing the modified DMEM (Ajinomoto); Yoshiko Naito and Noriyo Hayakawa for their technical assistance with the metabolome analysis (Department of Biochemistry, Keio University); Ryo Matoba and Hiroshi Iijima for their support with the RNA-seq analysis (DNA Chip Research, Inc.); and Hiroyuki Yamagishi, Keiko Uchida, and Kazuki Kodo for their fruitful discussions (Department of Pediatrics, Keio University). The authors also thank CiRA for the 253G4 and 201B7 cell lines, WiCell Research Institute for the H9 cell line, and Dr. David A. Elliott’s Laboratory for the NKX2-5^eGFP/w^ cell line. This work was supported by AMED grants (24bm1123010 to S.T. and JP22ama121016 to S.N.), KISTEC (to S.T.), JSPS KAKENHI (22KJ2668 to T.C.U.), Japanese Circulation Society (to S.T.), Keio University Doctorate Student Grant-in-Aid Program from the Ushioda Memorial Fund (to T.C.U.), Japan Heart Foundation Research Grant (to T.C.U.), and Heartseed Inc.

## Author contributions

S.T. conceptualized the study; T.C.U. and S.T. designed the study; T.C.U. performed and analyzed most of the experiments; Y.M.-U., M.K., S.N., and other co-authors contributed to specific experiments; T.C.U. and S.T. wrote the original draft and acquired the funding; and S.T., K.F., and M.I. supervised the study.

## Declaration of interests

T.C.U. and S.T. have a patent pending related to this work. K.F. is the co-founder and CEO of Heartseed Inc. S.T. is an advisor of Heartseed Inc. S.T. and K.F. own equity in Heartseed Inc. The remaining authors have no conflicts of interest to disclose.

## STAR★Methods

### Key resources table

**Table:** 

REAGENT or RESOURCE	SOURCE	IDENTIFIER
**Antibodies**		

anti-OCT3/4	Santa Cruz Biotechnology	Cat# sc-5279; RRID: AB_628051
anti-NANOG	abcam	Cat# ab21624; RRID:AB_446437
anti-PHGDH	Cell Signaling	Cat# 66350; RRID:AB_2737030
anti-PHGDH	Sigma-Aldrich	Cat# WH0026227M1; RRID:AB_1842961
anti-Cardiac Troponin T	Invitrogen	Cat# MA5-12960; RRID:AB_11000742
anti-Cardiac Troponin T	abcam	Cat# ab45932; RRID:AB_956386
anti-Sarcomeric α Actinin	Invitrogen	Cat# MA1-22863; RRID:AB_557426
anti-MLC2a	Synaptic Systems	Cat# 311 011; RRID:AB_887737
anti-MLC2v	abcam	Cat# ab79935; RRID:AB_1952220
anti-GAPDH	Thermo Fisher Scientific	Cat# AM4300; RRID:AB_2536381
anti-rabbit IgG Alexa Flour 488	Invitrogen	Cat# A11008; RRID:AB_143165
anti-mouse IgG Alexa Flour 546	Invitrogen	Cat# A11030; RRID:AB_2737024
anti-mouse IgG antibody	Thermo Fisher Scientific	Cat# A16072; RRID:AB_2534745
anti-cardiac Troponin T antibody conjugated to FITC	Miltenyi Biotec	Cat# 130-119-575; RRID:AB_2751735
anti-REA Control FITC	Miltenyi Biotec	Cat# 130-118-354; RRID:AB_2751490

**Chemicals, peptides, and recombinant proteins**		

Y-27632	FUJIFILM Wako Pure Chemical	Cat# 034-24024
CHIR99021	FUJIFILM Wako Pure Chemical	Cat# 034-23103
BMP4	R&D Synstems	Cat# 314-BP
NCT-503	Cayman Chemical	Cat# 19718
IWR-1	Sigma-Aldrich	Cat# 10161
D-PBS	FUJIFILM Wako Pure Chemical	Cat# 045-29795
AS103C	Ajinomoto	N/A
AS501	Ajinomoto	N/A
modified DMEM	Ajinomoto	N/A
XF DMEM medium pH 7.4	Agilent Technologies	Cat# 103575-100
XF 1.0 M Glucose Solution	Agilent Technologies	Cat# 103577-100
XF 200 mM Glutamine Solution	Agilent Technologies	Cat# 103579-100
B-27™ Supplement, minus insulin	Gibco	Cat# A1895601
Insulin-Transferrin-Selenium	Gibco	Cat# 41400045
MEM-α	Thermo Fisher Scientific	Cat# 12571-048
FBS	Biowest	Cat# S1560-500
Matrigel	Corning	Cat# 354230
iMatrix-511silk	Nippi	Cat# NP892-021
iMatrix-221	Nippi	Cat# NP892-061
Seahorse XF Plasma Membrane Permeabilizer	Agilent Technologies	Cat# 102504-100
Sucrose	FUJIFILM Wako Pure Chemical	Cat# 133-00845
Potassium dihydrogen phosphate	FUJIFILM Wako Pure Chemical	Cat# 167-04201
Magnesium chloride	NIPPON GENE	Cat# 310-90361
HEPES	Gibco	Cat# 15630080
EGTA	Bio Medical Science	Cat# BR-401201271
fatty acid-free BSA	FUJIFILM Wako Pure Chemical	Cat# 017-15141
Potassium hydroxide	Sigma-Aldrich	Cat# 221473
Adenosine diphosphate	Sigma-Aldrich	Cat# A5285
carbonyl cyanide-4-(trifluoromethoxy)phenylhydrazone	Sigma-Aldrich	Cat# C2920
Pyruvic acid	Sigma-Aldrich	Cat# 107360
Malic acid	Sigma-Aldrich	Cat# M0875
Succinic acid	Sigma-Aldrich	Cat# S3674
Ascorbic acid	Sigma-Aldrich	Cat# A5960
N,N,N',N'-tetramethyl-1,4-phenylenediamine dihydrochloride	Sigma-Aldrich	Cat# T7394
Rotenone	Sigma-Aldrich	Cat# R8875
Antimycin A	Sigma-Aldrich	Cat# R8674
Oligomycin	Sigma-Aldrich	Cat# O4876
MitoSOX indicators	Invitrogen	Cat# M36008
OptiMEM	Thermo Fisher Scientific	Cat# 31985070
Lipofectamine™ RNAi MAX Transfection Reagent	Thermo Fisher Scientific	Cat# 13778030
Mannitol	FUJIFILM Wako Pure Chemical	Cat# 133-00845
Methanol	FUJIFILM Wako Pure Chemical	Cat# 134-14521
L-methionine sulfone	FUJIFILM Wako Pure Chemical	Cat# 502-76641
2-morpholinoethanesulfonic acid	Dojindo	Cat# 341-01622
D-Glucose-^13^C_6_	ISOTEC	Cat# 389374
EmbryoMax® Nucleosides (100X)	Sigma-Aldrich	Cat# ES-008-D
SB431542	Cayman Chemical	Cat# 13031
PD173074	FUJIFILM Wako Pure Chemical	Cat# 160-26831
*N*-acetyl-L-cysteine	Sigma-Aldrich	Cat# A7250
Isoproterenol	Sigma-Aldrich	Cat# I6504
Cal-520, AM	AAT Bioquest	Cat# 21131
Hanks’ balanced salt solution	Thermo Fisher Scientific	Cat# 14025092
pluronic F-127	AAT Bioquest	Cat# 20053
ReadiUse™ probenecid	AAT Bioquest	Cat# 20062
TrypLE™ Select	Thermo Fisher Scientific	Cat# 12563-011
2.5g/L-Trypsin/1mmol/L-EDTA Solution	Nacalai	Cat# 35554-64
NuPAGE™ LDS Sample Buffer (4X)	Thermo Fisher Scientific	Cat# NP0007
NuPAGE™ Sample Reducing Agent (10X)	Thermo Fisher Scientific	Cat# NP0009
Protease Inhibitor Cocktail	Nacalai	Cat# 25955-24
Chemi-Lumi One L	Nacalai	Cat# 07880-54
SuperSignal™ West Femto Maximum Sensitivity Substrate	Thermo Fisher Scientific	Cat# 34095
4% paraformaldehyde	Muto Pure Chemicals	Cat# 33111
Triton™ X-100	Sigma-Aldrich	Cat# T9284
ImmunoBlock blocking solution	KAC	Cat# CTKN001
Hoechst 33342	Thermo Fisher Scientific	Cat# H3570

**Critical commercial assays**		

ReliaPrep™ RNA Cell Miniprep System	Promega	Cat# Z6012
Superscript™ First Strand Synthesis System	Invitrogen	Cat# 11904018
TruSeq Stranded mRNA Library Prep Kit	Illumina	Cat# 20020595
Chromium 3’ v3 Chemistry Kit	10x Genomics	Cat# PN-1000075
NovaSeq S4 Reagent Kit	Illumina	Cat# 20027466
Seahorse XF Cell Mito Stress Test Kit	Agilent Technologies	Cat# 103015-100
Seahorse XF Real-Time ATP Rate Assay Kit	Agilent Technologies	Cat# 103592-100
Seahorse XFe24 Extracellular Flux Assay Kits	Agilent Technologies	Cat# 102340-100
NuPAGE™ Bis-Tris Mini Protein Gels, 4–12%, 1.0–1.5 mm	Thermo Fisher Scientific	Cat# NP0321BOX
iBlot™ 2 Transfer Stacks, PVDF, mini	Thermo Fisher Scientific	Cat# IB24002
Cell Cycle Analysis Kit	abcam	Cat# ab287852
Annexin V-FITC Apoptosis Staining / Detection Kit	abcam	Cat# ab14085

**Deposited data**		

hPSCs and hPSC-CMs RNA-seq	This study	GSE290355
Day 1, Day 3, Day 5, and Day 9 scRNA-seq	This study	GSE289789

**Experimental models: Cell lines**		

253G4	CiRA^50^	N/A
201B7	CiRA^51^	N/A
H9	WiCell^52^	N/A
NKX2-5^eGFP/w^	David A Elliott’s laboratory^52^	N/A

**Oligonucleotides**		

Silencer Negative Control siRNA	Thermo Fisher Scientific	Cat# AM4611
siRNA targeting PHGDH	Thermo Fisher Scientific	Cat# 133785
ACTN2 (Hs00153809)	Thermo Fisher Scientific	Cat# 4331182
ATP2A2 (Hs01564013)	Thermo Fisher Scientific	Cat# 4331182
EBF1 (Hs01092694)	Thermo Fisher Scientific	Cat# 4331182
ISL1 (Hs00158126)	Thermo Fisher Scientific	Cat# 4331182
KCNH2 (Hs00542479)	Thermo Fisher Scientific	Cat# 4331182
KCNQ1 (Hs00923522)	Thermo Fisher Scientific	Cat# 4331182
MESP1 (Hs00251489)	Thermo Fisher Scientific	Cat# 4331182
MYH6 (Hs01101425)	Thermo Fisher Scientific	Cat# 4331182
MYH7 (Hs01110632)	Thermo Fisher Scientific	Cat# 4331182
MYL2 (Hs00166405)	Thermo Fisher Scientific	Cat# 4331182
MYL7 (Hs01085598)	Thermo Fisher Scientific	Cat# 4331182
PHGDH (Hs01106329)	Thermo Fisher Scientific	Cat# 4331182
PLN (Hs01848144)	Thermo Fisher Scientific	Cat# 4331182
POU5F1 (Hs04260367)	Thermo Fisher Scientific	Cat# 4331182
T (Hs00610080)	Thermo Fisher Scientific	Cat# 4331182
TBX1 (Hs00962558)	Thermo Fisher Scientific	Cat# 4331182
TNNI1 (Hs00913333)	Thermo Fisher Scientific	Cat# 4331182
TNNI3 (Hs00165957)	Thermo Fisher Scientific	Cat# 4331182
TNNT2 (Hs00943911)	Thermo Fisher Scientific	Cat# 4331182
GAPDH (Hs02758991)	Thermo Fisher Scientific	Cat# 4331182

**Software and algorithms**		

Seahorse Wave Desktop Software	Agilent Technologies	https://www.agilent.com/en/product/cell-analysis/real-time-cell-metabolic-analysis/xf-software/seahorse-wave-desktop-software-740897
Seahorse XF Cell Mito Stress Test Report Generators	Agilent Technologies	https://www.agilent.com/en/product/cell-analysis/real-time-cell-metabolic-analysis/xf-software/seahorse-xf-cell-mito-stress-test-report-generators-740899
Seahorse XF Real-Time ATP Rate Assay Report Generators	Agilent Technologies	https://www.agilent.com/en/product/cell-analysis/real-time-cell-metabolic-analysis/xf-software/seahorse-xf-real-time-atp-rate-assay-report-generators-740902
FlowJo software	Tomy Digital Biology	https://www.flowjo.com/solutions/flowjo/downloads
MasterHands	Sugimoto et al.^53^	N/A
ImageJ	NIH	https://imagej.nih.gov/ij/
GraphPad Prism 10	GraphPad	N/A
Design & Analysis 2 (DA2) software	Thermo Fisher Scientific	N/A
StrandNGS software ver 4.0	Agilent Technologies	N/A
Cell Ranger software ver 6.1.2	10x Genomics	https://www.10xgenomics.com/support/software/cell-ranger/downloads
R ver 4.2.2	CRAN	https://www.R-project.org/.
Seurat ver 4.1.2 and ver 5.0.1	Hao et al.^54^	https://satijalab.org/seurat/
CellBender ver 0.3.2	Fleming et al.^55^	https://github.com/broadinstitute/CellBender
metascape	Zhou et al.^56^	https://metascape.org/
ClusterProfiler ver 4.14.4	Yu et al.^57^ Wu et al.^58^ Xu et al.^59^ Yu.^60^	https://github.com/YuLab-SMU/clusterProfiler
dplyr ver 1.1.2	Wickham et al.^61^	https://github.com/tidyverse/dplyr
tools ver 4.2.2	CRAN	http://www.R-project.org/
monocle3 ver 1.3.4	Trapnell et al.^62^ Qiu et al.^63^ Cao et al.^64^	https://github.com/cole-trapnell-lab/monocle3
ComplexHeatmap ver 2.14.0	Gu et al.^65^ Gu et al.^66^	https://github.com/jokergoo/ComplexHeatmap
circlize ver 0.4.16	Gu et al.^67^	https://github.com/jokergoo/circlize
RColorBrewer ver 1.1.3	CRAN	https://CRAN.R-project.org/package=RColorBrewer
ggprism ver 1.0.5	CRAN	https://CRAN.R-project.org/package=ggprism
ggplot2 ver 3.4.2	Wickham H.^68^	https://github.com/tidyverse/ggplot2
Matrix ver 1.5.4	CRAN	https://CRAN.R-project.org/package=Matrix
org.Hs.eg.db ver 3.16.0	Carlson et al.^69^	https://bioconductor.org/packages/org.Hs.eg.db/

**Other**		

Multiwell Plate 6 well	IWAKI	Cat# 3810-006N
Multiwell Plate 12 well	IWAKI	Cat# 3815-012
Multiwell Plate 24 well	IWAKI	Cat# 3820-024

### Experimental model and study participant details

#### Cell lines

The human induced pluripotent stem cell lines (253G4 and 201B7) were obtained from the Center for iPS Cell Research and Application (CiRA), Kyoto University.^50^^,^^51^ The hESC line (H9) was provided by WiCell.^70^ The hESC line (NKX2-5^eGFP/w^) was provided by Dr. David A Elliott’s laboratory, Murdoch Children Research Institute.^52^ All cell lines used in this study originated from female donors. hPSCs were cultured in a humidified 5% CO_2_ incubator, at 37°C, and routinely tested for mycoplasma contamination. hPSCs were maintained on Matrigel™ (354230, Corning)- or iMatrix-511silk (NP892-021, Nippi)-coated plates, in animal-free and chemically defined hPSC maintenance medium (StemFit™ AS103C, Ajinomoto).^71^^,^^72^

### Method details

#### Maintenance of hPSCs

The cells were routinely passaged every 4–7 days. Briefly, after being washed with Dulbecco’s phosphate-buffered saline (D-PBS; 045-29795, FUJIFILM Wako Pure Chemical), the cells were dissociated using TrypLE™ Select (12563-011, Thermo Fisher Scientific), at 37°C. The dissociated cells were collected in StemFit™ AS103C with 5 μM of CultureSure® Y-27632 (034-24024, FUJIFILM Wako Pure Chemical). Following centrifugation (300×*g* for 4 min), supernatant aspiration, and addition of StemFit™ AS103C with 5 μM CultureSure® Y-27632, the cells were counted using Vi-CELL XR (Beckman Coulter). The cells were seeded onto Matrigel™- or iMatrix-511silk-coated plates. The medium was replaced every other day with AS103C.

#### CM differentiation from hPSCs

hPSCs were seeded onto Matrigel™- or iMatrix-511silk-coated 6 well plates (67-0324-22, IWAKI) and differentiated into CMs by modifying the method described in previous reports.^73^^,^^74^ Briefly, on Day 0, upon reaching 90%–100% confluence, the cells were rinsed with D-PBS and incubated with modified DMEM (Ajinomoto) containing 11.1 mM glucose supplemented with 2% B27™ Supplement minus insulin (A1895601, Thermo Fisher Scientific), 6 μM CHIR99021 (034-23103, FUJIFILM Wako Pure Chemical), and 1 ng/mL BMP4 (314-BP, R&D Systems), for 24 h (mesoderm induction). On Day 1, the cells were rinsed with D-PBS and incubated with modified DMEM containing B-27™ Supplement minus insulin with 5 μM NCT-503 (19718, Cayman Chemical) or vehicle control (DMSO) (D8418, Sigma). On Day 3, the cells were rinsed with D-PBS and incubated with modified DMEM supplemented with B27™ Supplement minus insulin and 5 μM IWR-1 (I0161, Sigma). On Day 5, the cells were rinsed with D-PBS and incubated in modified DMEM with B27™ Supplement minus insulin. On Day 7, the cells were incubated in modified DMEM with B27™ Supplement minus insulin and 1× insulin-transferrin-selenium (41400045, Gibco). On Day 9, the cells were washed with D-PBS and dissociated using 2.5g/L-Trypsin/1mmol/L-EDTA Solution (35554-64, Nacalai) and then resuspended in MEM-α (12571-048, Thermo Fisher Scientific) supplemented with 5% fetal bovine serum (FBS) (S1560-500, Biowest) and 2 mM sodium pyruvate (S8636, Sigma). The cells were then seeded onto iMatrix-221 (NP892-061, Nippi)-coated plates. On Day 13, the cells were washed with D-PBS and incubated with glucose- and glutamine-free medium supplemented with 4 mM lactate, StemFit™ medium AS501 (Ajinomoto) for metabolic selection.^22^ After four days of metabolic selection, the cells were collected and resuspended in MEM-α supplemented as previously described and then seeded onto iMatrix-221-coated 6 well plates. hPSC-CMs obtained on Day 30 were used in the experiments. The 253G4, 201B7, H9, NKX2-5^eGFP/w^ cell lines were used for experiments in which metabolic features of hPSCs and hPSC-CMs were evaluated using RNA-seq. hiPSCs (253G4) were used for the other experiments.

#### Immunocytochemistry

Cells were rinsed with D-PBS and fixed with 4% paraformaldehyde (33111, Muto Pure Chemicals) for 20 min. The cells were treated with 0.1% Triton™ X-100 (T9284, Sigma) diluted in D-PBS for 5 min, rinsed with D-PBS, and then treated with ImmunoBlock blocking solution (CTKN001, KAC). The cells were treated with primary antibodies diluted in blocking solution at the dilutions recommended by the manufacturer, at 4°C overnight. The cells were rinsed with D-PBS and treated with secondary antibodies diluted in a blocking solution, at 4°C overnight. The cells were treated with 5 μg/mL Hoechst 33342 (H3570, Thermo Fisher Scientific) at room temperature, for 10 min. The cells were imaged using a BZ-X710 microscope (Keyence). Immunofluorescence staining of cells was performed using the following primary antibodies: anti-OCT-3/4 (sc-5279; 1:50, Santa Cruz Biotechnology), anti-NANOG (ab21624, 1:50, Abcam), anti-PHGDH (66350, 1:200, Cell Signaling Technology), anti-cardiac Troponin T (MA5-12960, 1:200, Thermo Fisher Scientific or ab45932, 1:200, Abcam), anti-sarcomeric α-actinin (MA1-22863, 1:200, Invitrogen), anti-MLC2a (C156F5 311011, 1:200, Synaptic Systems), and anti-MLC2v (ab79935, 1:200, Abcam); and the following secondary antibodies: anti-rabbit or anti-mouse IgG conjugated with Alexa Flour™ 488 or 546 (1:200, all from Thermo Fisher Scientific).

#### Flow cytometry analysis

For cardiac Troponin T expression, the cells were fixed with 4% paraformaldehyde for 20 min, permeabilized with 0.1% Triton™ X, and then stained with an anti-cardiac Troponin T antibody conjugated to FITC (130-119-575, 1:50, Miltenyi Biotec). The cells were analyzed using a Gallios Flow Cytometer (Beckman Coulter) and FlowJo software (Tomy Digital Biology). The negative control consisted of cells stained with anti-REA Control FITC (130-118-354, 1:50, Miltenyi Biotec).

#### Contractile analysis of hiPSC-CMs

Contractile analysis was performed on Day 30 hiPSC-CMs using an SI8000 live-cell motion imaging system (Sony Corporation) and analyzed by modifying the method described in a previous report.^75^ The contraction peaks were analyzed in terms of beating rate, force, contraction duration, and relaxation duration.

#### Drug responses of hiPSC-CMs using FDSS/μCELL

Cryopreserved hiPSC-CMs were thawed and cultured for approximately 1 week in a culture medium containing 2.5% FBS and sodium pyruvate in MEM-α. Drug responses were analyzed by modifying the method described in a previous report.^76^ Briefly, on the day of the assay, hiPSC-CMs were incubated in 5 mM Cal-520®, AM (21131, AAT Bioquest) in Hanks’ balanced salt solution (14025092, Thermo Fisher Scientific) supplemented with pluronic F-127® (20053, AAT Bioquest) and probenecid (20062, AAT Bioquest) at 37°C for 1 h. Isoproterenol (I6504; Sigma-Aldrich) was added to the culture medium at specific concentrations. Finally, the expression of calcium transient was measured using FDSS/μCELL (Hamamatsu Photonics K.K.).

#### RNA extraction and quantitative RT-PCR analysis

Total RNA samples were isolated using a ReliaPrep™ RNA Cell Miniprep System (Z6012, Promega), according to the manufacturer’s instructions. cDNA was synthesized using the Superscript™ First-Strand Synthesis System (11904018, Invitrogen). Quantitative RT-PCR was performed using the QuantStudio™ 7 Pro Real-Time PCR System (Applied Biosystems), with the following TaqMan® probes (Thermo Fisher Scientific): *ACTN2* (Hs00153809), *ATP2A2* (Hs01564013), *EBF1* (Hs01092694), *ISL1* (Hs00158126), *KCNH2* (Hs00542479), *KCNQ1* (Hs00923522), *MESP1* (Hs00251489), *MYH6* (Hs01101425), *MYH7* (Hs01110632), *MYL2* (Hs00166405), *MYL7* (Hs01085598), *PHGDH* (Hs01106329), *PLN* (Hs01848144), *POU5F1* (Hs04260367), *T* (Hs00610080), *TBX1* (Hs00962558), *TNNI1* (Hs00913333), *TNNI3* (Hs00165957), and *TNNT2* (Hs00943911). The mRNA levels were normalized to those of *GAPDH* (Hs02758991). The data were analyzed using Design & Analysis 2 (DA2) software (Thermo Fisher Scientific).

#### RNA-seq

RNA was extracted from hPSCs or hPSC-CMs on Day 30, using the ReliaPrep™ RNA Cell Miniprep System. The total RNA obtained from each sample was subjected to sequencing library construction using the TruSeq® Stranded mRNA Library Prep Kit (20020595, Illumina, San Diego, CA, USA), according to the manufacturer’s protocols. Equally pooled libraries of the samples were sequenced on a NovaSeq 6000 System (Illumina), in 101-bp paired-end reads. Sequencing adaptors, low-quality reads, and bases were trimmed using the Trimmomatic-0.39 tool. Sequence reads were aligned to the human reference genome (hg38) using STAR 2.7.10b. The aligned reads were subjected to downstream analyses using the StrandNGS software (version 4.0; Agilent Technologies). The read counts allocated to each gene and transcript (RefSeq Database 2016.12.01) were quantified using the transcripts-per-million method. DEGs were considered significant at fold-change>10.

#### scRNA-seq sample and library generation

hiPSCs were differentiated into CMs using the method described above. The cells were treated with NCT-503 or DMSO from Day 1 to Day 3 and collected on Days 1, 3, 5, and 9. Following that, 8000 cells were prepared to a concentration of 1,000 cells/μL and loaded into the Chromium Controller (10× Genomics) and single-cell cDNA libraries were generated using a Chromium 3′ v3 Chemistry Kit (PN-1000075, 10× Genomics). Libraries were sequenced on a NovaSeq 6000 System, using the NovaSeq S4 Reagent Kit (20027466, Illumina).

#### scRNA-seq data processing and clustering

Raw FASTQ files were processed for each sample using the Cell Ranger software (version 6.1.2, 10× Genomics), against the Cell Ranger GRCh38 human reference genome. Raw mapped counts were used as the input for data processing with the Seurat R package (version 4.1.2, 5.0.1).^54^ To exclude ambient RNA contamination, the data were processed using CellBender.^55^ We removed cells with detected genes <2000, detected counts <2000 and >30000, and mitochondrial gene content >15%. Following the filtering step, we normalized the read counts using the ‘NormalizeData’ function (10,000 default scale factor), separately for each dataset. We used the ‘FindVariableFeatures’ function to identify highly variable features for downstream analysis and integrated them using the ‘FindIntegrationAnchors’ and ‘IntegrateData’ functions. The integrated data were used for dimensionality reduction and cluster detection. We performed linear regression using the ‘ScaleData’ function and a linear dimensionality reduction using the ‘RunPCA’ function. Twenty principal components were used for downstream graph-based, supervised clustering into distinct populations using the ‘FindClusters’ function and Uniform Manifold Approximation and Projection (UMAP) dimensionality reduction was performed to project the cell population onto two dimensions using the ‘RunUMAP’ function. DEGs were detected using the ‘FindMarkers’ function (log_2_fc.threshold>0.25 and p_val_adj<0.05). After we retrieved the data for each cell type, we used the ‘FindVariableFeatures’ function to identify highly variable features for downstream analysis. We then used the ‘ScaleData’, ‘RunPCA’, ‘FindClusters’, ‘RunUMAP’, and ‘FindMarkers’ functions on the subsets of every cell cluster. To assess and visualize the gene expression patterns across the identified clusters in our scRNA-seq dataset, we used the DotPlot function in the Seurat package. DEGs were identified according to adjusted *p*-values (p_val_adj<0.05) and then subjected to GO enrichment analysis using Metascape^56^ or ClusterProfiler (version 4.14.4).^57^

#### Single-cell trajectory and pseudotime analysis

For the construction of single-cell developmental trajectories and discovering trajectory transitions, we performed single-cell trajectory and pseudotime analysis using monocle3 package (version 1.3.4).^62^^,^^63^^,^^64^ We converted the Seurat object into a SingleCellExperiment object and ordered cells along the pseudotime trajectory with the function of order_cells. Monocle3 identifies branch points that describe significant divergences in the cellular state automatically. Thus, the pseudotime trajectory can be used to infer major differentiation paths based on the ramification in gene expression. To identify key genes along the developmental trajectory during cardiac differentiation, we extracted DEGs with q-value < 0.01 via spatial autocorrelation analysis using Moran’s I statistics. A heatmap was applied for visualization of gene expressions along the developmental trajectory using ComplexHeatmap (version 2.14.0).^65^^,^^66^

#### Metabolome analysis using [U-^13^C]-labeled glucose

The cells were cultured in modified DMEM with 11.1 mM D-Glucose-^13^C_6_ (389374, ISOTEC) supplemented with B27™ Supplement minus insulin for 12 h. The cells were rinsed with ice-cold 5% (w/v) mannitol (133-00845, FUJIFILM Wako Pure Chemical) and collected with methanol (134-14521, FUJIFILM Wako Pure Chemical) that contained internal standards of 200 μM L-methionine sulfone (502-76641, FUJIFILM Wako Pure Chemical) and 200 μM 2-morpholinoethanesulfonic acid (341-01622, Dojindo). The metabolites were analyzed using an Agilent CE-TOF-MS System equipped with an Agilent G7100A CE instrument and an Agilent 6530 Q-TOF LC/MS system (Agilent Technologies). Raw data were processed using MasterHands (Human Metabolome Technologies).^53^ The metabolites were identified by matching the m/z and corrected migration times to those in our standard library. The absolute concentration was quantified based on the ratio of the peak areas of each metabolite to those of the internal standard compounds. Values were corrected using the total protein weights.

#### Measurement of OCR

hiPSCs were seeded onto iMatrix-511silk-coated XFe24 cell culture microplates (Agilent Technologies). After 24 h of mesoderm induction, the cells were analyzed in glucose- and glutamine-depleted XF-DMEM (103575-100, Agilent Technologies) supplemented with 11.1 mM glucose (103577-100, Agilent Technologies) and 4 mM glutamine (103579-100, Agilent Technologies) with NCT-503 or DMSO. The OCR was measured using a Seahorse XF Cell Mito Stress Test Kit (103015-100, Agilent Technologies). The cells were analyzed using an XFe24 Extracellular Flux Analyzer (Agilent Technologies), according to the manufacturer’s instructions. The OCR was normalized using cell count numbers and analyzed using Seahorse XF Cell Mito Stress Test Report Generators (Agilent Technologies).

#### Calculation of ATP production rate

hiPSCs were seeded onto iMatrix-511silk-coated XFe24 cell culture microplates and differentiated into CMs, as described above. hiPSC-CMs were seeded onto iMatrix-221-coated XFe24 cell culture microplates. On each measurement day, the medium was replaced with glucose- and glutamine-depleted XF-DMEM supplemented with 11.1 mM glucose and 4 mM glutamine. Extracellular acidification rate and OCR were measured using the Seahorse XF Real-Time ATP Rate Assay Kit (103592-100, Agilent Technologies). The cells were analyzed using an Xfe24 Extracellular Flux Analyzer, according to the manufacturer’s instructions. Extracellular acidification rate and OCR were normalized to cell counts and analyzed using Seahorse XF Real-Time ATP Rate Assay Report Generators (Agilent Technologies).

#### Measurement of mitochondrial electron transport chain complex activity

hiPSCs were seeded onto iMatrix-511silk-coated XFe24 cell culture microplates. After mesoderm induction for 24 h, the cells were analyzed using a non-ionic mannitol and sucrose-based buffer (MAS) with NCT-503 or DMSO. MAS buffer was composed of 70 mM sucrose (196-00015, FUJIFILM Wako Pure Chemical), 220 mM mannitol, 10 mM potassium dihydrogen phosphate (167-04201, FUJIFILM Wako Pure Chemical), 5 mM magnesium chloride (310-90361, Nippon Gene), 2 mM HEPES (15630080, Gibco), 1 mM EGTA (BR-401201271, Bio Medical Science), and 0.2% fatty acid-free bovine serum albumin (017-15141, FUJIFILM Wako Pure Chemical).^31^ MAS buffer was adjusted to a pH of 7.2 using potassium hydroxide (221473, Sigma-Aldrich). Seahorse XF Plasma Membrane Permeabilizer (1 nM; 102504-100, Agilent Technologies), adenosine diphosphate (4 mM; A5285, Sigma-Aldrich), carbonyl cyanide-4-(trifluoromethoxy)phenylhydrazone (0.25 μM; C2920, Sigma-Aldrich), and oxidizable substrates of interest were added to the MAS buffer. Oxidizable substrates and specific inhibitors to each mitochondrial complex used in the assay are listed as follows: complex I-mediated substrates 10 mM pyruvic acid (107360, Sigma-Aldrich) and 1 mM malic acid (M0875, Sigma-Aldrich); complex II-mediated substrates 10 mM succinic acid (S3674, Sigma-Aldrich); complex IV-mediated substrates 10 mM ascorbic acid (A5960, Sigma-Aldrich) and 100 μM N,N,N',N'-tetramethyl-1,4-phenylenediamine dihydrochloride (T7394, Sigma-Aldrich); complex I inhibitor 2 μM rotenone (R8875, Sigma-Aldrich); and complex III inhibitor 2 μM antimycin A (A8674, Sigma-Aldrich). The cells were analyzed using an XFe24 Extracellular Flux Analyzer, according to the manufacturer’s instructions. All data analyses were performed using the Seahorse Wave Desktop Software (Agilent Technologies).

#### Measurement of glucose consumption and lactate secretion

Cells were incubated in modified DMEM containing 11.1 mM glucose supplemented with 2% B27™ Supplement minus insulin and NCT-503 or DMSO. Supernatant samples were collected and analyzed using BioProfile FLEX2 (Nova Biomedical).

#### Measurement of mitochondrial superoxide

MitoSOX™ indicators (M36008, Invitrogen) were used to detect mitochondrial ROS, according to the manufacturer’s instructions. Cells were incubated with modified DMEM containing 11.1 mM glucose supplemented with 2% B27™ Supplement minus insulin and NCT-503 or DMSO, for three hours. The cells were analyzed using a Gallios Flow Cytometer, with FlowJo software.

#### Apoptosis assays

Cells treated with NCT-503 or DMSO were analyzed using an Annexin V Apoptosis Detection Kit (ab14085, Abcam) according to the manufacturer’s instructions. Cells were stained with Annexin V-TITC and propidium iodide, and analyzed using a Gallios Flow Cytometer, with FlowJo software.

#### Cell cycle assays

Cells treated with NCT-503 or DMSO were analyzed using a Cell Cycle Analysis Kit (ab287852, Abcam) according to the manufacturer’s instructions. Cells were fixed by 70% cold ethanol, stained with nuclear dye, and analyzed using a Gallios Flow Cytometer, with FlowJo software.

#### PHGDH knockdown in hiPSCs

hiPSCs were transfected with Silencer Negative Control siRNA (AM4611, Thermo Fisher Scientific) or 100 nM PHGDH-targeting siRNA (133785, Thermo Fisher Scientific), using Lipofectamine™ RNAiMAX Transfection Reagent (13778030, Thermo Fisher Scientific) and Opti-MEM™ (31985070, Thermo Fisher Scientific), according to the manufacturer’s instructions. The medium was changed 24 h after transfection, and the cells were incubated for an additional 48 h. To measure OCR, each transfected cell was seeded onto iMatrix-511silk-coated XFe24 cell culture microplates and analyzed using an XFe24 Extracellular Flux Analyzer according to the manufacturer’s instructions. The OCR was normalized using cell count numbers and analyzed using Seahorse XF Cell Mito Stress Test Report Generators.

#### Western blot

Lysates were prepared by means of homogenization of cells in cell lysis solution [25% NuPAGE™ LDS Sample Buffer (4×) (NP0007, Thermo Fisher Scientific), 10% NuPAGE™ Sample Reducing Agent (10×) (NP0009, Thermo Fisher Scientific), 65% ultrapure water, and Protease Inhibitor Cocktail (25955-24, Nacalai)]. Ten μg of protein was loaded into NuPAGE™ 4%–12% Bis-Tris gel (NP0321BOX, Thermo Fisher Scientific). After transfer to iBlot2™ PVDF Mini Stacks (IB24002, Thermo Fisher Scientific), immunodetection was performed using antibodies specific to PHGDH (WH0026227M1, 1:1000, Sigma-Aldrich) and GAPDH (AM4300, 1:25000, Thermo Scientific). Protein expression was visualized using a horseradish peroxidase-conjugated secondary antibody (A16072, 1:25000, Thermo Scientific) and Chemi-Lumi One L (07880-54, Nacalai) or SuperSignal™ West Femto Maximum Sensitivity Substrate (34095, Thermo Fisher Scientific). Images were obtained using an iBright™ FL1000 Imaging System (Thermo Fisher Scientific) and band intensities were quantified using ImageJ.

### Quantification and statistical analysis

All statistical analyses were performed using GraphPad Prism 10 (GraphPad Software Inc). Values are presented as mean ± SD or mean ± SEM. Differences between groups were examined for statistical significance using Student’s *t* test or ANOVA. Statistical significance was set at *p* < 0.05: ∗*p* < 0.05 and ∗∗*p* < 0.01.
