# Supplementary material for: Serine synthesis pathway regulates cardiac differentiation from human pluripotent stem cells
Source: iScience. 2025 Jun 7;28(7):112843. doi: 10.1016/j.isci.2025.112843 (PMC12256312; doi:10.1016/j.isci.2025.112843)
Supplement: Document S1. Figures S1–S5 [file mmc1.pdf]

iScience, Volume *28*

Supplemental information

Serine synthesis pathway regulates cardiac differentiation from human pluripotent stem cells

Tomohiko C. Umei, Shugo Tohyama, Yuika Morita-Umei, Manami Katoh, Seitaro Nomura, Kotaro Haga, Takako Hishiki, Tomomi Matsuura, Hidenori Tani, Yusuke Soma, Otoya Sekine, Masatoshi Ohno, Masashi Nakamura, Taijun Moriwaki, Yoshikazu Kishino, Keiichi Fukuda, and Masaki Ieda

Figure S1

A

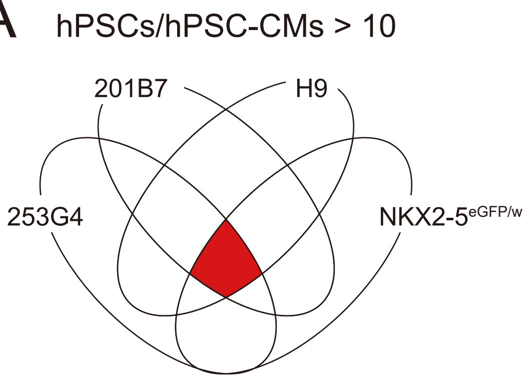

B

BCAT1	*Branched chain amino acid transaminase 1*
HMOX1	*Heme oxygenase 1*
PSAT1	*Phosphoserine aminotransferase 1*
TKT	*Transketolase*
PRPS2	*Phosphoribosyl pyrophosphate synthetase 2*
PHGDH	*Phosphoglycerate dehydrogenase*
LPCAT1	*Lysophosphatidylcholine acyltransferase 1*

C

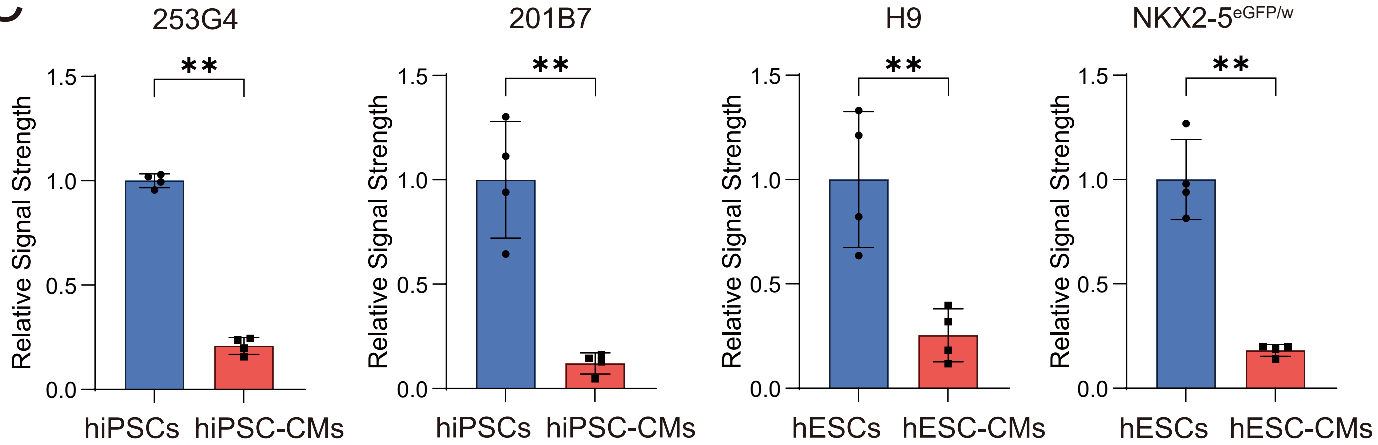

D

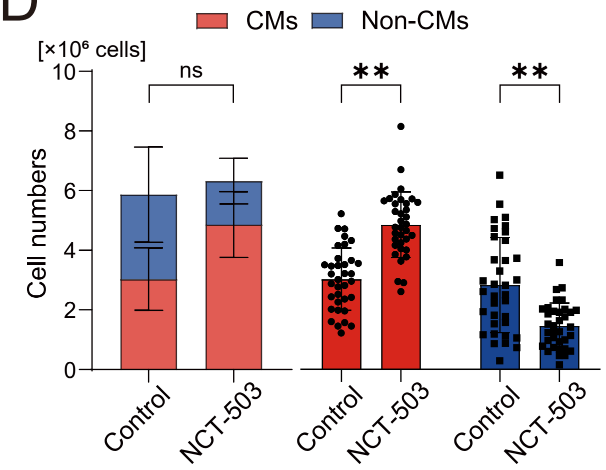

Figure S2

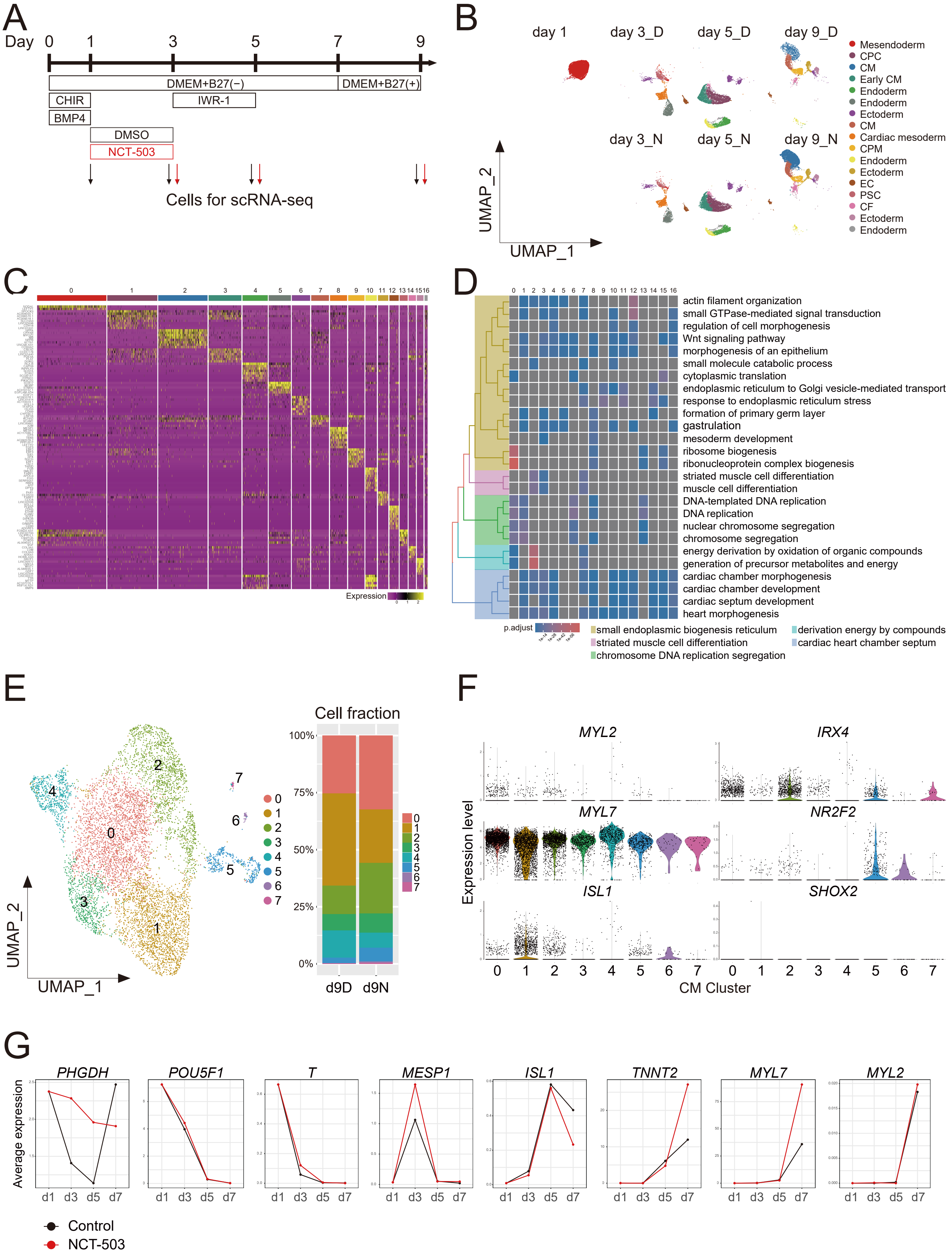
A
Day 0 1 3 5 7 9
DMEM+B27(−)
DMEM+B27(+)
CHIR
BMP4
IWR-1
DMSO
NCT-503
Cells for scRNA-seq
B
day 1
day 3_D
day 5_D
day 9_D
day 3_N
day 5_N
day 9_N
UMAP_2
UMAP_1
Mesendoderm
CPC
CM
Early CM
Endoderm
Endoderm
Ectoderm
CM
Cardiac mesoderm
CPM
Endoderm
Ectoderm
EC
PSC
CF
Ectoderm
Endoderm
C
0 1 2 3 4 5 6 7 8 9 10 11 12 13 14 15 16
Expression
D
0 1 2 3 4 5 6 7 8 9 10 11 12 13 14 15 16
actin filament organization
small GTPase-mediated signal transduction
regulation of cell morphogenesis
Wnt signaling pathway
morphogenesis of an epithelium
small molecule catabolic process
cytoplasmic translation
endoplasmic reticulum to Golgi vesicle-mediated transport
response to endoplasmic reticulum stress
formation of primary germ layer
gastrulation
mesoderm development
ribosome biogenesis
ribonucleoprotein complex biogenesis
striated muscle cell differentiation
muscle cell differentiation
DNA-templated DNA replication
DNA replication
nuclear chromosome segregation
chromosome segregation
energy derivation by oxidation of organic compounds
generation of precursor metabolites and energy
cardiac chamber morphogenesis
cardiac chamber development
cardiac septum development
heart morphogenesis
p.adjust
small endoplasmic biogenesis reticulum
striated muscle cell differentiation
chromosome DNA replication segregation
derivation energy by compounds
cardiac heart chamber septum
E
UMAP_2
UMAP_1
0 1 2 3 4 5 6 7
Cell fraction
100%
75%
50%
25%
0%
d9D d9N
F
MYL2
IRX4
MYL7
NR2F2
ISL1
SHOX2
Expression level
0 1 2 3 4 5 6 7
CM Cluster
G
PHGDH
POU5F1
T
MESP1
ISL1
TNNT2
MYL7
MYL2
Average expression
d1 d3 d5 d7
Control
NCT-503

Figure S3

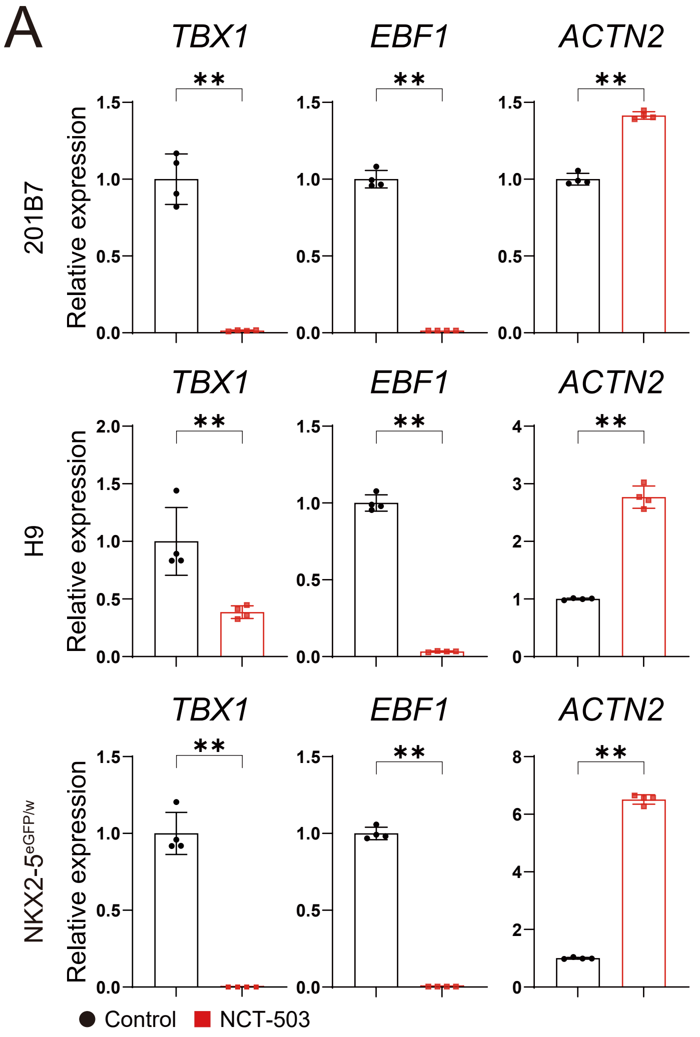
A
TBX1
EBF1
ACTN2
201B7
H9
NKX2-5eGFP/w
Relative expression
**
Control
NCT-503

Figure S4

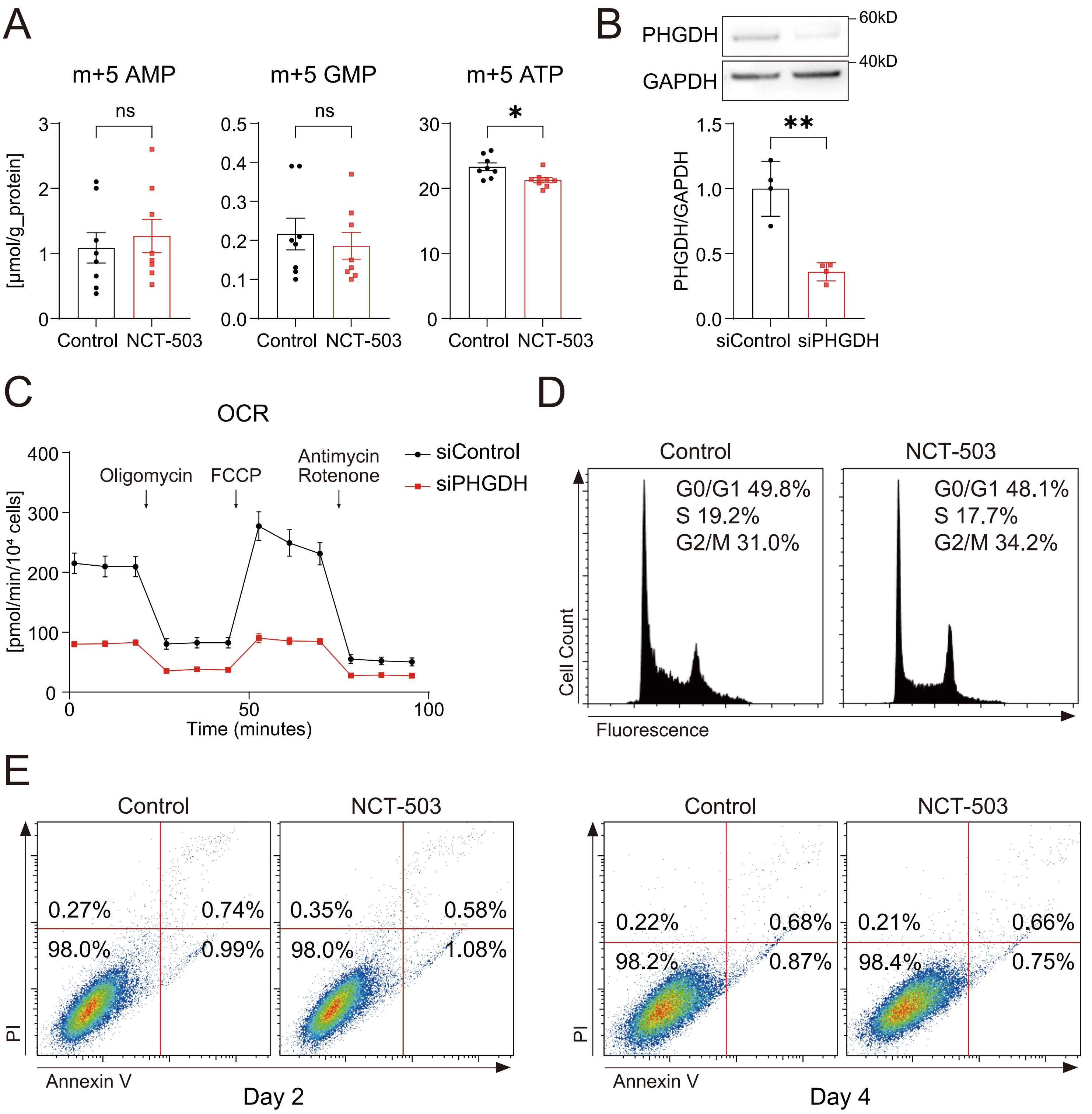
A
m+5 AMP
m+5 GMP
m+5 ATP
[μmol/g_protein]
ns
ns
*
Control
NCT-503
B
PHGDH
GAPDH
60kD
40kD
PHGDH/GAPDH
**
siControl
siPHGDH
C
OCR
Oligomycin
FCCP
Antimycin
Rotenone
siControl
siPHGDH
[pmol/min/10⁴ cells]
Time (minutes)
D
Control
NCT-503
G0/G1 49.8%
S 19.2%
G2/M 31.0%
G0/G1 48.1%
S 17.7%
G2/M 34.2%
Cell Count
Fluorescence
E
Control
NCT-503
0.27%
0.74%
98.0%
0.99%
0.35%
0.58%
98.0%
1.08%
0.22%
0.68%
98.2%
0.87%
0.21%
0.66%
98.4%
0.75%
PI
Annexin V
Day 2
Day 4

Figure S5

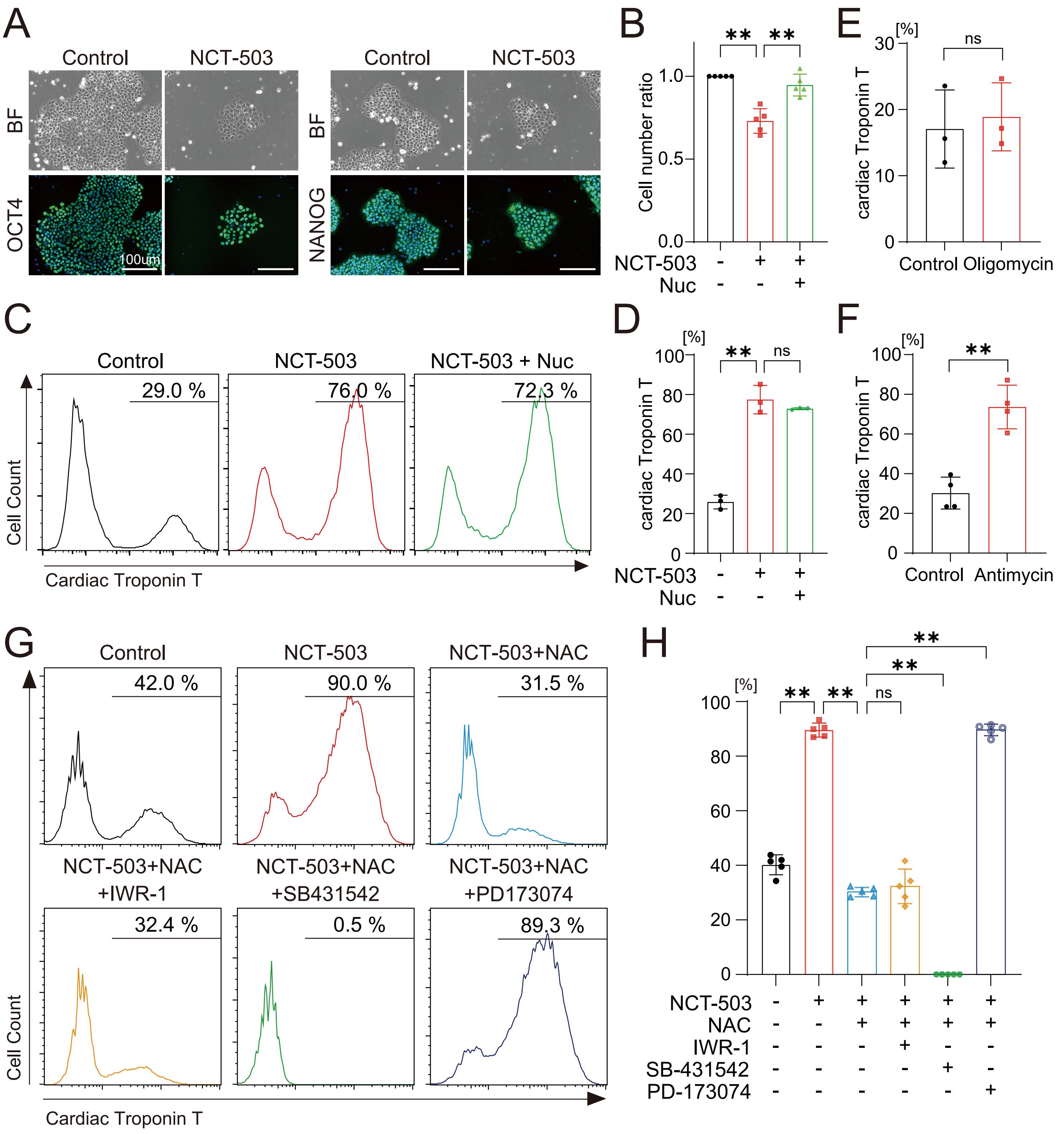
A
Control
NCT-503
Control
NCT-503
BF
OCT4
100um
BF
NANOG
B
Cell number ratio
NCT-503
Nuc
E
cardiac Troponin T
[%]
ns
Control
Oligomycin
C
Control
29.0 %
NCT-503
76.0 %
NCT-503 + Nuc
72.3 %
Cell Count
Cardiac Troponin T
D
cardiac Troponin T
[%]
ns
NCT-503
Nuc
F
cardiac Troponin T
[%]
Control
Antimycin
G
Control
42.0 %
NCT-503
90.0 %
NCT-503+NAC
31.5 %
NCT-503+NAC
+IWR-1
32.4 %
NCT-503+NAC
+SB431542
0.5 %
NCT-503+NAC
+PD173074
89.3 %
Cell Count
Cardiac Troponin T
H
[%]
ns
NCT-503
NAC
IWR-1
SB-431542
PD-173074

Supplemental Figure Legends

Figure S1. PHGDH Is Highly Expressed in hiPSCs and hESCs, related to Figure 1.

(A) Gene expression analysis of hPSCs and hPSC-CMs. Venn diagram demarks similarities and differences among four hPSCs/hPSC-CMs groups. **(B)** Genes commonly upregulated in the hPSC groups. **(C)** Relative amounts of PHGDH in hPSCs and hPSC-CMs on Day 30 quantified by western blotting. Signal strengths were standardized using those of GAPDH. Student's t-test was performed for each analysis ($n = 3$). **(D)** Quantification of cells harvested on Day 9, with control or NCT-503 treatment from Day 1 to Day 3. Student's t-test was performed for each analysis ($n = 35$).
$^{**}p < 0.01$. Data are shown as mean ± SD. Experimental repeats were completely independent.
CM, cardiomyocyte; hESC, human embryonic stem cell; hESC-CM, human embryonic stem cell-derived cardiomyocyte; hPSC, human pluripotent stem cell; hPSC-CM, human induced pluripotent stem cell-derived cardiomyocyte.

Figure S2. Detailed scRNA-seq Analysis under SSP Inhibition, related to Figure 3.

(A) Schematic illustration of the hPSC-CM differentiation protocol and timing of cell collection for scRNA-seq analysis. **(B)** The UMAP plot of scRNA-seq data displays individual cells by cell type under each condition. **(C)** Heatmap illustrating the expression of top markers in each cluster. **(D)** Tree plot illustrating Gene Ontology (GO) enrichment for all clusters and the correlation between GO terms. **(E)** UMAP plot (left) of *TNNT2*-positive CM populations on Day 9, displaying individual cells by cell type. Bar plot (right) shows the distribution of cells in each sample. **(F)** Violin plot showing the expression of genes indicative of developmental and subtype CM markers. **(G)** Line graph showing the change of gene expression under each condition at differentiation timing.
BMP4, bone morphogenetic protein 4; CF, cardiac fibroblast; CM, cardiomyocyte; CPC, cardiac progenitor cell; CPM, cardiopharyngeal mesoderm; DMEM, Dulbecco’s Modified Eagle’s Medium; DMSO, dimethylsulfoxide; EC, endothelial cell; hPSC-CM, human pluripotent stem cell-derived cardiomyocyte; PSC, pluripotent stem cell; scRNA-seq, single-cell RNA sequencing; UMAP, Uniform Manifold Approximation and Projection.

Figure S3. Inhibition of PHGDH Decreased CPM Markers, related to Figure 4.

(A) qPCR analysis of CPM and CM markers on Day 9 with control or NCT-503 treatment from Day 1 to Day 3 in 201B7, H9, and NKX2-5$^{eGFP/w}$. mRNA expression

under control conditions was defined as the control value. Student's t-test was performed for each analysis ($n = 4$).
**$p < 0.01$. Data are shown as mean ± SD. Experimental repeats were completely independent.

Figure S4. Inhibition of PHGDH Impairs Mitochondrial Respiration, but Does Not Affect Cell Cycle and Cell Viability, related to Figure 5. (A) [U-^{13}C]-labeled metabolites related to nucleotide synthesis, under control or NCT-503 treatment condition. Student's t-test was performed ($n = 8$). **(B)** Representative western blot images of PHGDH and GAPDH in hiPSCs, after treatment with siCtrl or PHGDH siRNA. The graph shows the relative amount of PHGDH quantified by western blotting. Signal strengths were standardized using those of GAPDH. Student's t-test was performed ($n = 4$). **(C)** Representative mitochondrial OCR, after treatment with siCtrl or PHGDH siRNA, as measured using a flux analyzer ($n = 5$). **(D)** Representative image of cell cycle analysis on Day 3 with control or NCT-503 treatment conditions from Day 1 to Day 3. **(E)** Representative image of apoptosis analysis on Day 2 (left) and Day 4 (right) with control or NCT-503 treatment conditions from Day 1 to Day 3.
*$p < 0.05$; **$p < 0.01$. Data are shown as mean ± SD (**B**) or mean ± SEM (**A and C**). Experimental repeats were completely independent (**A and B**) or simultaneous well-replicates (**C**).
AMP, Adenosine monophosphate; ATP, adenosine triphosphate; GMP, guanosine monophosphate; OCR, oxygen consumption rate; hiPSC, human induced pluripotent stem cell; PI, propidium iodide; siRNA, small interfering RNA.

Figure S5. Rescue Experiments of Nucleotide Supplementation and Signaling Pathway Modulators, related to Figure 6. (A) Representative images of immunocytochemistry of undifferentiated state markers, after treatment with control of NCT-503 for four days. **(B)** The graph displays the cell number ratio of harvested hiPSCs, under control, NCT-503, or NCT-503+Nuc conditions. One-way analysis of variance (ANOVA) with Dunnett's test was performed ($n = 5$). **(C)** Representative flow cytometric analysis of cardiac Troponin T expression on Day 9 with control, NCT-503, or NCT-503+Nuc condition from Day 1 to Day 3. **(D)** The graph displays the proportion of cardiac Troponin T-positive cells under each condition. One-way ANOVA with Dunnett's test was performed ($n = 3$). (**E**) Proportion of cardiac Troponin T-positive cells on Day 9 with control or Oligomycin (5 ng/mL) treatment from Day 1 to Day 3. Student's t-test was performed for each analysis ($n = 3$). **(F)** Proportion of cardiac Troponin T-positive cells on Day 9 with control or Antimycin A (5 nM) treatment from

Day 1 to Day 3. Student's t-test was performed for each analysis (*n* = 3). **(G)** Representative flow cytometric analysis of cardiac Troponin T expression on Day 9 with control, NCT-503, NCT-503+NAC, or NCT-503+NAC+each signaling pathway modulator (5 μM IWR-1, 5 μM SB431542, or 50 nM PD173074) treatment from Day 1 to Day 3. **(H)** Proportion of cardiac Troponin T-positive cells under each condition. One-way ANOVA with Dunnett's test was performed (*n* = 5).

*$p < 0.05$; **$p < 0.01$. Data are shown as mean ± SD. Experimental repeats were completely independent.

BF, bright field; DMSO, dimethylsulfoxide; hiPSC, human induced pluripotent stem cell; NAC, *N*-acetyl-L-cysteine, Nuc, nucleosides.
